# Development and Validation of an AI‐Driven System for Automatic Literature Analysis and Molecular Regulatory Network Construction

**DOI:** 10.1002/advs.202405395

**Published:** 2024-10-07

**Authors:** Jia Li, Hailin Zhang, Jiamin Wang, Mei Deng, Zhiyong Li, Wei Jiang, Kejin Xu, Lianlian Wu, Zehua Dong, Jun Liu, Qianshan Ding, Honggang Yu

**Affiliations:** ^1^ Department of Gastroenterology Renmin Hospital of Wuhan University Wuhan Hubei 430060 P. R. China; ^2^ Hubei Key Laboratory of Digestive System Renmin Hospital of Wuhan University Wuhan Hubei 430060 P. R. China; ^3^ Hubei Provincial Clinical Research Center for Digestive Disease Minimally Invasive Incision Renmin Hospital of Wuhan University Wuhan Hubei 430060 P. R. China; ^4^ Engineering Research Center for Artificial Intelligence Endoscopy Interventional Treatment of Hubei Province Wuhan Hubei 430060 P. R. China; ^5^ Nursing Department of Renmin Hospital of Wuhan University Wuhan Hubei 430060 P. R. China

**Keywords:** artificial intelligence, automatic literature analysis, molecular regulatory networks

## Abstract

Decoding gene regulatory networks is essential for understanding the mechanisms underlying many complex diseases. GENET is developed, an automated system designed to extract and visualize extensive molecular relationships from published biomedical literature. Using natural language processing, entities and relations are identified from a randomly selected set of 1788 scientific articles, and visualized in a filterable knowledge graph. The performance of GENET is evaluated and compared with existing methods. The named entity recognition model has achieved an overall precision of 94.23% (4835/5131; 93.56–94.84%), recall of 97.72% (4835/4948; 97.27–98.10%), and an F1 score of 95.94%. The relation extraction model has demonstrated an overall precision of 91.63% (2593/2830; 90.55–92.59%), recall of 89.17% (2593/2908; 87.99–90.25%), and an F1 score of 90.38%. GENET significantly outperforms existing methods in extracting molecular relationships (P < 0.001). Additionally, GENET has successfully predicted WNT family member 4 regulates insulin‐like growth factor 2 via signal transducer and activator of transcription 3 in colon cancer. With RNA sequencing data and multiple immunofluorescence, the authenticity of this prediction is validated, supporting the promising feasibility of GENET.

## Graphical abstract

GENET is an automated system that extracts and visualizes extensive detailed molecular relationships from published biomedical literature with superior performance compared to existing models, and it has preliminarily demonstrated its effectiveness in exploring potential molecular mechanisms.

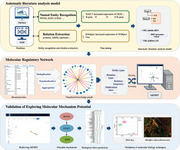

## 1 Introduction

Gene and protein functions are realized within a complex multilevel system, where interactions between molecules are essential for numerous cellular processes and are determinants of cell fate.^[^ ^1^ ^]^ With the growing availability of “omics” data, an increasing number of disease‐related genes have been identified. Understanding and deciphering the molecular mechanisms underlying diseases such as cancer is critical, as it can provide valuable insights for diagnosis and treatment. Therefore, decoding molecular interaction networks is crucial in uncovering the molecular basis of complex diseases and understanding the pharmacological effects of drugs.^[^ ^2^ ^]^

High‐throughput screening methods are commonly employed to investigate the mechanisms of specific molecules by analyzing their potential downstream targets. However, these approaches are not only costly but also require high‐quality samples and advanced bioinformatics analysis. Additionally, high‐throughput technologies may not always provide clear insights into the regulatory relationships between molecules.^[^ ^3^, ^4^ ^]^ Alternatively, researchers often turn to traditional methods, which involve reviewing published literature to generate scientific hypotheses, followed by validation using molecular biology techniques. However, the information on molecular interactions is scattered across numerous databases and articles. As the volume of biomedical literature continues to grow exponentially, manually collecting and managing this information becomes increasingly time‐consuming and labor‐intensive, with redundant data from multiple publications potentially hindering research progress.^[^ ^5^ ^]^

The advent of artificial intelligence (AI), particularly natural language processing (NLP) technology, has enabled rapid analysis of electronic health records and bioinformatics data, allowing for the exploration of disease mechanisms.^[^ ^6^, ^7^ ^]^ Databases such as INTACT,^[^ ^8^ ^]^ BIOGRID,^[^ ^9^, ^10^ ^]^ and STRING^[^ ^11^, ^12^, ^13^ ^]^ have been developed to address these challenges by providing researchers with structured, high‐quality, and detailed information on gene or protein interactions, whether previously known or predicted. The development and utilization of these databases have significantly advanced molecular biology and human disease research. However, most of these databases do not specify regulatory patterns or directions, which are crucial for understanding cellular functional behavior. Moreover, they often lack real‐time updates, making it difficult for researchers to stay up to date with the latest research findings. For example, in a previous study, we reported that WNT family member 4 (WNT4) is overexpressed in colorectal cancer.^[^ ^14^ ^]^ However, we were still unable to fully elucidate the molecular mechanisms driving the progression of colorectal cancer due to the limitations mentioned above.

In this study, we introduce GENET, an AI‐based automatic literature analysis model designed for constructing molecular regulatory networks. Unlike existing methods,^[^ ^8^, ^9^, ^10^, ^11^, ^12^, ^13^, ^15^, ^16^, ^17^, ^18^ ^]^ GENET is a real‐time automated system that not only provides detailed information on the specific mechanisms and directions of molecular interactions, but also visually presents the biological effects of these molecules as reported in the literature. This tool enables researchers to quickly grasp the underlying mechanisms and functions of molecules, and their possible positions within signaling networks. GENET's performance was evaluated using a test set and compared with existing databases and tools. Additionally, we demonstrated GENET's potential and practicality by exploring the molecular mechanisms of WNT4, using sequencing data and immunofluorescence techniques. This work not only alleviates the burden of literature retrieval and organization for researchers, thereby enhancing their efficiency, but also introduces a novel technology to progressively unravel the complex regulatory networks between molecules, ultimately contributing to the advancement of precision medicine.

## 2 Results

### 2.1 Performance of the Automatic Literature Analysis Model

In the automatic literature analysis model, the named entity recognition (NER) model achieved an overall precision of 94.23% (4835/5131; 93.56–94.84%), recall of 97.72% (4835/4948; 97.27–98.10%), and an F1 score of 95.94%, Gene entities exhibited the highest performance, with precision, recall, and F1 scores of 96.27% (2582/2682; 95.48–96.92%), 98.59% (2582/2619; 98.06–98.98%), and 97.42%, respectively. The entity relation extraction (RE) model demonstrated overall precision, recall, and F1 scores of 86.94% (2630/3025; 85.69–88.09%), 90.44% (2630/2908; 89.32–91.46%), and 88.66%, respectively, with the entity abbreviation relation achieving the highest scores: precision of 90.19% (331/367, 86.72–92.83%), recall of 95.66% (331/346, 92.97–97.35%), and an F1 score of 92.84%. When combining the NER model results, the RE model's overall precision, recall, and F1 scores improved to 91.63% (2593/2830; 90.55–92.59%), 89.17% (2593/2908; 87.99–90.25%), and 90.38%, respectively. The entity abbreviation relation again performed best, with precision, recall, and F1 scores of 92.66% (328/354, 89.46–94.94%), 94.80% (328/346, 91.93–96.69%), and 93.72%, respectively. Detailed performance metrics for the automatic literature analysis model are presented in **Table** **1** and Figure S1 (Supporting Information).

**Table 1:** Performance of the automatic literature analysis model.

	Precision [n/N, 95% CI]	Recall [n/N, 95% CI]	F1 score
The named entity recognition model of the automatic literature analysis model			
Gene	96.27% (2582/2682; 95.48–96.92%)	98.59% (2582/2619; 98.06–98.98%)	97.42%
Signal pathway	95.59% (455/476, 93.35–97.10%)	98.70% (455/461, 97.19–99.40%)	97.12%
Cancer	95.35% (717/752, 93.60–96.64%)	97.82% (717/733, 96.49–98.65%)	96.57%
Biological function	88.53% (1081/1221; 86.62–90.20%)	95.24% (1081/1135; 93.84–96.33%)	91.76%
Overall	94.23% (4835/5131; 93.56–94.84%)	97.72% (4835/4948; 97.27–98.10%)	95.94%
The entity relation extraction model of the automatic literature analysis model			
Promotes	88.47% (959/1084, 86.43–90.24%)	90.13% (959/1064; 88.19–91.78%)	89.29%
Inhibits	86.27% (710/823, 83.75–88.45%)	90.45% (710/785, 88.19–92.31%)	88.31%
Upstream	88.25% (278/315, 84.22–91.35%)	92.67% (278/300, 89.15–95.11%)	90.41%
Abbreviation	90.19% (331/367, 86.72–92.83%)	95.66% (331/346, 92.97–97.35%)	92.84%
Function	80.73% (352/436, 76.77–84.16%)	85.23% (352/413, 81.48–88.33%)	82.92%
Overall	86.94% (2630/3025; 85.69–88.09%)	90.44% (2630/2908; 89.32–91.46%)	88.66%
The entity relation extraction model of integrated automatic literature analysis model			
Promotes	92.69% (951/1026, 90.93–94.13%)	89.38% (951/1064; 87.39–91.09%)	91.00%
Inhibits	91.94% (696/757, 89.78–93.67%)	88.66% (696/785, 86.25–90.69%)	90.27%
Upstream	92.33% (277/300, 88.76–94.83%)	92.33% (277/300, 88.76–94.83%)	92.33%
Abbreviation	92.66% (328/354, 89.46–94.94%)	94.80% (328/346, 91.93–96.69%)	93.72%
Function	86.77% (341/393, 83.06–89.77%)	82.57% (341/413, 78.62–85.92%)	84.62%
Overall	91.63% (2593/2830; 90.55–92.59%)	89.17% (2593/2908; 87.99–90.25%)	90.38%

When excluding “function” entities and their corresponding relations, the NER model's overall precision, recall, and F1 scores were 95.60% (3762/3935; 94.91–96.20%), 98.66% (3762/3813; 98.24–98.98%), and 95.72%, respectively. The RE model achieved overall precision, recall, and F1 scores of 82.20% (1473/1792; 80.36–83.90%), 90.20% (1473/1633; 88.66–91.55%), and 86.01%, respectively. The integrated model, combining NER results, showed overall precision, recall, and F1 scores of 87.97% (1455/1654; 86.31–89.45%), 89.10% (1455/1633; 87.50–90.52%), and 88.53%, respectively. Performance details of the automatic literature analysis model, excluding biological functions, are provided in Table S1 and Figure S2 (Supporting Information).

### 2.2 Performance of GENET in Molecular Relation Extraction

Among the 100 molecular relations randomly selected and verified through expert literature review, GENET demonstrated superior performance compared to existing models, with a recall of 97.00% (97/100, 91.55–98.97%). GENIE3, one of the existing methods, showed comparable performance to GENET [89.00% (89/100, 81.37–93.75%) versus 97.00% (97/100, 91.55–98.97%), P = 0.057]. The performance of the other seven methods was significantly lower (P < 0.001 for all). In addition to providing confidence assessments, GENET offers detailed insights into the directions and mechanisms of molecular interactions. Comparative results are presented in **Table** **2** and Figure S3 (Supporting Information).

**Table 2:** Performance of GENET in molecular relation extraction compared with that of existing methods.

Methods	Specific modes of molecular interactions	Confidence level assessment	Recall
GENET	Equipped	Equipped	97.00% (97/100, 91.55–98.97%)
BioGRID^a)^	Equipped	Equipped	13.00% (13/100, 7.76–20.98%)^*^
GeneMANIA^b)^	Not equipped	Equipped	29.00% (29/100, 21.01–38.54%)^*^
GEPI^c)^	Equipped	Equipped	75.00% (75/100, 65.70–82.45%)^*^
STRING^d)^	Not equipped	Equipped	37.00% (37/100, 28.18–46.78%)^*^
GENIE3^[^ ^15^ ^]^	Not equipped	Not equipped	89.00% (89/100, 81.37–93.75%)
GRNBoost2^[^ ^16^ ^]^	Not equipped	Not equipped	66.00% (66/100, 56.28–74.54%)^*^
KBoost^[^ ^17^ ^]^	Not equipped	Not equipped	49.00% (49/100, 39.42–58.65%)^*^
STGRNS^[^ ^18^ ^]^	Not equipped	Not equipped	75.00% (75/100, 65.70–82.45%)^*^

* Significant difference between the target groups and GENET (*p* < 0.001);

^a)^ https://thebiogrid.org/;

^b)^ https://genemania.org/;

^c)^ https://gepi.coling.uni-jena.de/;

^d)^ http://string.embl.de/.

### 2.3 Performance of GENET in Exploring Molecular Mechanisms

**Figure 1:**
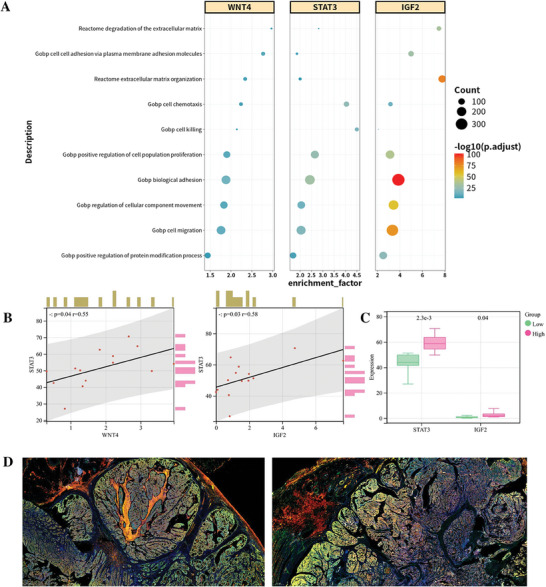
GENET's Performance in Exploring Molecular Mechanisms. A) Example results from the functional enrichment analysis. B) Pearson correlation analysis of the transcriptome data. C) Rank‐sum test results of the transcriptome data after dividing the samples into high and low WNT4 expression groups. D) Multiplex immunofluorescence results show WNT4 in red, IGF2 in green, and STAT3 in yellow.

A search for WNT4 and insulin‐like growth factor 2 (IGF2) on the GENET platform identified signal transducer and activator of transcription 3 (STAT3) as the most direct intermediary molecule, suggesting that WNT4 promotes or activates IGF2 by activating STAT3 (Video S1, Supporting Information). Following filtration, bioinformatics functional enrichment analysis revealed associations of WNT4, STAT3, and IGF2 with 340 biological functions, including cell adhesion, cell migration, and cell proliferation. Pearson correlation analysis of transcriptome data from 14 patients with colon cancer indicated strong positive correlations between WNT4 and STAT3, and between STAT3 and IGF2. Rank‐sum tests, after categorizing the 14 transcriptome samples into high and low WNT4 expression groups, revealed significant differences in STAT3 and IGF2 expression levels between the high and low WNT4 groups. Additionally, multiplex immunofluorescence analyses of pathological sections from 25 patients with colon cancer confirmed co‐expression of WNT4, STAT3, and IGF2. Details of the functional enrichment analysis, transcriptome validation, and multiplex immunofluorescence results are presented in **Figures** **1** and S4,S5 (Supporting Information).

Video S1 (Supporting Information). Demonstration of GENET in Action.

## 3 Discussion

In this study, we developed an AI‐based automatic literature analysis model and molecular regulatory network system, named GENET, to explore and decipher potential molecular mechanisms. GENET significantly outperforms existing methods by enabling real‐time, automated extraction and visualization of gene interactions from published biomedical literature. This system offers dynamic visualization of molecular interaction networks, thereby enhancing researchers' efficiency in data capture and overall productivity. By identifying key biological targets or markers, GENET provides new directions and targets for future biomedical research, contributing to the advancement of precision medicine.

Deciphering the intricate interrelations among molecules is essential for deepening our understanding of biological systems. However, the exponential growth of medical literature presents a significant challenge for researchers trying to stay on top of the latest scientific developments.^[^ ^19^, ^20^ ^]^ Traditionally, investigating molecular mechanisms relied heavily on experimental techniques such as polymerase chain reaction (PCR), western blotting (WB), and high‐throughput sequencing.^[^ ^21^, ^22^ ^]^ While effective, these methods are often time‐consuming, labor‐intensive, and constrained by their data throughput, making it difficult to keep pace with the rapid rate of discovery. Therefore, it is crucial to automate the extraction of vital information from the vast literature, allowing researchers to efficiently identify the most critical molecular relationships.^[^ ^2^ ^]^

GENET addresses this need by providing a powerful tool that facilitates the deeper exploration of molecular mechanisms. Processing each article in under 50 ms, GENET significantly reduces the need for researchers to manually search and summarize the literature, freeing up valuable time for innovation and critical analysis. By automatically analyzing and aggregating articles into an easy‐to‐navigate interface, GENET not only streamlines the research process but also ensures that important advancements and insights are not missed. The AI‐driven extraction and visualization capabilities of this model greatly enhance the accessibility and interpretability of complex data, overcoming the limitations of human capacity to process and analyze large volumes of information.^[^ ^23^ ^]^

This model offers researchers a valuable resource, enabling them to maintain a comprehensive understanding of complex molecular interactions and mechanisms without the need to individually sift through numerous publication. GENET provides an automated and comprehensive approach to extracting and visualizing complex molecular relationships from a vast corpus of literature. Researchers can easily search or zoom into the knowledge graph using the search bar and filter options, helping them to prioritize interactions for further exploration and guiding experimental design. GENET rapidly identifies potential relationships and mechanisms that might otherwise take longer to discover through experimental methods alone, significantly accelerating the research and development process. It allows researchers to navigate the complexities of molecular biology with unprecedented ease and precision, opening new pathways for scientific discovery and hypothesis generation.

GENET not only meticulously delineates the specific mechanisms and directions of interactions between molecules but also extracts the biological effects of these molecules directly from the literature, complementing predictions made by traditional bioinformatics techniques.^[^ ^24^ ^]^ Existing methods designed to elucidate molecular interactions typically focus on the interaction data itself, without exploring the detailed mechanisms or biological effects described in the literature.^[^ ^25^ ^]^ GENET bridges this gap by integrating detailed molecular relationships (such as promotion, inhibition, and upstream effects) from various publications, constructing a comprehensive knowledge graph of molecular interactions. This approach provides a more holistic view of molecular dynamics by combining the “why” and “how” of interactions with their observed outcomes. Unlike traditional databases that rely on bioinformatics techniques to predict molecular biological functions, GENET enriches the scope of the available data by narrowing the gap between the empirical biological insights found in public databases and those documented in the literature. This results in a more complete and insightful resource that supports a deeper exploration of molecular mechanisms by researchers.

A significant highlight of this work is our innovative use of GENET to discover and validate a new pathway in colon cancer, specifically, the promotion of IGF2 by WNT4 through the activation of STAT3. Traditionally, unraveling the mechanisms involving WNT4 and IGF2 in colon cancer would require extensive literature reviews or costly high‐throughput screening technologies. By utilizing GENET to swiftly and automatically sift through and analyze the existing literature, we were able to quickly hypothesize and confirm that STAT3 serves as the key intermediary in the interaction between WNT4 and IGF2. This significantly accelerated the pace of discovery and hypothesis validation,^[^ ^26^, ^27^, ^28^, ^29^ ^]^ demonstrating the unprecedented capabilities of AI in biomedical research. Moreover, understanding STAT3's role in mediating the effects of WNT4 on IGF2 opens new avenues for therapeutic intervention, potentially leading to more effective treatments for patients. This finding holds practical significance for developing targeted and personalized treatments for colon cancer. Our group plans to conduct more detailed experiments to further clarify how WNT4 regulates IGF2 via STAT3.

Similar molecular mechanisms may exist across different diseases; for instance, the human EGF‐like receptor 2 was initially considered an important prognostic and predictive marker in breast cancer, but with technological advances, gene amplification, and overexpression have also been discovered in gastric, ovarian, prostate, and lung cancers.^[^ ^30^, ^31^ ^]^ Notably, GENET facilitates the exploration of gene roles across different diseases, which will provide researchers with new research inspiration. Not limited to this, GENET allows for the creation of a personalized knowledge graph, which can be filtered based on interaction type, frequency, cancer type, and other criteria (including but not limited to publication time, reputation of the journal, etc.), making it applicable to a wide range of basic research tasks (Video S1, Supporting Information). Based on these functions of GENET, analyzing the knowledge graph can help summarize the key mechanisms common or distinct in different diseases, and it aids in gaining a deeper understanding of the genomes and pathogenesis of patients with various types of cancer, identifying potential drug targets and combinations, and accelerating the discovery and development of new drugs.^[^ ^32^ ^]^

Importantly, GENET serves as a foundational framework that bridges existing knowledge in biomedicine and drug development.^[^ ^33^ ^]^ By integrating virtual screening and molecular docking techniques, GENET enhances the capabilities of computer‐aided drug design, enabling a more detailed depiction of the potential biological effects of drugs on downstream pathways. Additionally, by incorporating metagenomic and single‐cell sequencing data, GENET offers researchers deeper insights into the interactions between cells and between diseases and microorganisms.^[^ ^34^, ^35^ ^]^ Genes typically do not function in isolation, but operate cooperatively within complex networks. Biological function modules, which represent groups of genes that work together within specific biological processes or pathways, are crucial for understanding how different genes collaborate to achieve specific biological functions. Emerging algorithms, such as the BMCL algorithm, can simulate the flow of information within these networks, facilitating the identification of functional modules or gene clusters.^[^ ^36^ ^]^ Through cluster analysis, it may be possible to discover previously unknown gene modules associated with specific biological functions or diseases. We are committed to developing these functional modules to make GENET's capabilities more comprehensive, with related work to be released soon.

However, our study does present some limitations. Firstly, the varying quality of data within the literature can introduce noise into the molecular relationships extracted by GENET. Future efforts should focus on automatically assessing the confidence levels of molecular relationships within the literature and integrating this with external experimental validation data, sequencing data, and other sources. Secondly, while we have identified and validated a potential mechanism involving WNT4, STAT3, and IGF2 in colon cancer using sequencing data and immunofluorescence techniques, additional technologies such as WB and PCR are required to verify their regulatory relationships.^[^ ^37^ ^]^

## 4 Conclusion

In conclusion, this study introduces GENET, an automatic literature analysis model, and molecular regulatory network designed to extract and visualize molecular interactions from a vast corpus of literature. GENET outperforms existing methods, holding significant promise in identifying potential molecular relationships and mechanisms, staying current with the latest developments in the literature, and pinpointing potential molecular targets.

## 5 Experimental Section

### Datasets

Using WNT4 as a case study, a strong association between WNT4 and IGF2 were predicted by integrating deep learning with pathological section analysis.^[^ ^38^ ^]^ The predictive details of molecules closely associated with WNT4 expression at the pathological level are provided in the Supporting Information and Table S2 (Supporting Information). However, the specific interaction between these two molecules has not been previously elucidated. To address this gap, GENET was developed, a tool designed for automatic literature analysis and visualization of molecular regulatory networks, to explore the regulatory mechanism between WNT4 and IGF2.

To ensure a comprehensive collection of publications and to gather sufficient intermolecular regulatory data for model construction, all relevant literature abstracts pertaining were retrieved to the WNT and IGF families in cancer from the PubMed database. A random subset of 1788 abstracts was selected and divided into training, test, and validation sets based on individual publications to develop the literature analysis model. The entirety of the collected literature was used to construct a visual molecular regulatory network.

### Development and Validation of the Automatic Literature Analysis Model

**Figure 2:**
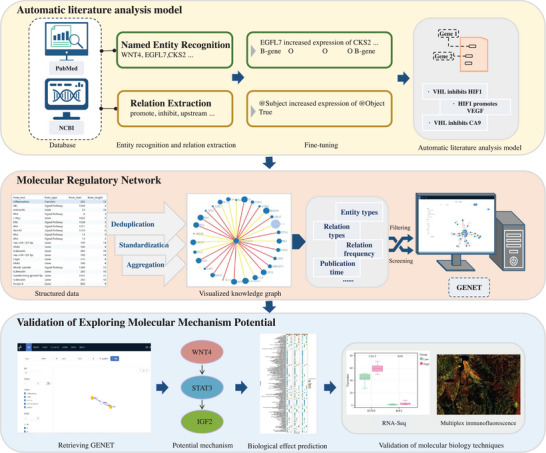
Development Framework and Validation Steps of GENET. Automatic Literature Analysis Model: Literature from PubMed, combined with the NCBI database, was annotated and used as ground truth for named entity recognition (NER) and relation extraction (RE). The BIO tagging method was employed to annotate entity boundaries and types (including genes, signaling pathways, cancers, etc.) in the text for the NER task. The RE model identified target entities (e.g., EGFL7 as “@Subject” and CKS2 as “@Object”) and determined the relationships between them (such as promotion, inhibition, upstream relations) based on their context within the text. Through detailed text analysis, the model discerned the semantics and context of entities, enabling the extraction of valuable relation triples. Molecular Regulatory Network: The relation triples were deduplicated, standardized, and aggregated into a visual knowledge graph that can be filtered and screened by relation type, frequency, and publication time. Validation of Molecular Mechanism Exploration: IGF2 and WNT4 were retrieved from the GENET website to investigate their interrelationship. The identified molecular pathways were validated using bioinformatics techniques, transcriptome data, and multiplex immunofluorescence.

The automatic literature analysis model comprises two independent and parallel‐processed modules: NER and RE. A single expert annotated a randomly selected set of literature abstracts using the Label Studio platform, establishing a gold standard for model training. The BIO tagging method was employed to mark the boundaries and types of entities for NER tasks within the text. The RE model identified target entities and recognized relationships between them based on the context. Entity annotation included four categories: genes, signaling pathways, cancer, and biological function. The GENE database from the National Center for Biotechnology Information (NCBI) (https://www.ncbi.nlm.nih.gov/) served as a reference for gene entity annotation. Relation annotation was used to link detailed interactions between entities, including promotion, inhibition, upstream, abbreviation, and function relations. The framework for developing the automatic literature analysis model is illustrated in **Figure** **2**.

For the NER task, the Flair natural language processing framework was combined with the pre‐trained PubMedBERT‐large embeddings. Flair excels at capturing contextual relations in the text, while PubMedBERT‐large, pre‐trained on the PubMed database, provides rich biomedical data contextual features, making it particularly sensitive to biomedical terms. Initially, PubMedBERT‐large was used to obtain pre‐trained embeddings for each word and Flair to obtain character‐level contextual representations, which help capture subtle spelling and morphological information. These embeddings were then merged and an additional linear layer was applied to integrate the vectors of entities, forming an enhanced, context‐sensitive word embedding. To capture long‐distance dependencies between words, the embeddings were input into a Recurrent Neural Network (RNN), which provided a context‐informed representation for each word or token. To further enhance the NER model's performance, the output was fed into a Conditional Random Field (CRF) layer. The CRF layer learns and predicts the most likely tag sequence by establishing a conditional probability model, thus significantly improving the model's sequence labeling capabilities.

**Figure 3:**
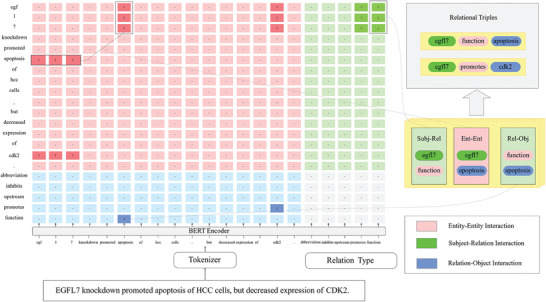
The architecture of the interaction map. The interaction graph illustrates the model's internal attention mechanism, comprising entity‐entity interactions (indicated by red coordinates) and entity‐relationship interactions (subjects indicated by green coordinates and objects by blue coordinates). This simulation of interactions between entities and their relationships enhances the extraction of relation triples from the text. HCC: Hepatocellular carcinoma.

For the RE task, it began by using nltk. tokenize to intelligently segment the annotated literature, thereby shortening the maximum length of sentences input into the model. the PubMedBERT‐large model was used, based on Huggingface, as the pre‐trained model, and combined it with the Unirel framework, aiming to achieve RE through unified representation and interaction.^[^ ^39^ ^]^ In Unirel, input text was transformed into unified interactions by building an interaction map. This map uses the Transformer's self‐attention mechanism to simulate interactions between entities and between entities and relations, thus more effectively extracting relational triples from the text. The architecture of the interaction map is presented in **Figure** **3**. The loss function used was Binary Cross Entropy Loss, which encourages the model to accurately identify associated entities and relations in the text. To fully account for sentence structure and the complex relationships between entities, we separated sentence encoding and relation prediction, allowing the model to independently process text and predict relations. Additionally, extra linear layers were introduced to merge the encodings of sentences and relations, further enhancing the model's performance.

The NER and RE models were converted from dynamic to static graphs and then into TensorRT models. By performing int8 quantization on some data and deploying the models using the Triton Inference Server framework, we enabled the model to process a standard‐length literature abstract in less than 50 ms. This approach maintained high accuracy while significantly boosting inference speed.

The initial RE model did not account for entity types, a critical factor since the relation types between different entities are contextually constrained (e.g., there cannot be an abbreviation relation between “gene” entities and “function” entities). Therefore, to develop an automatic literature analysis model capable of simultaneously identifying both entity types and relation categories, it began by considering the positions of entities within sentences. The outputs from both the NER and RE models were then integrated to create the final automatic literature analysis model. This approach not only improved the accuracy of the RE model but also ensured the uniqueness of nodes within the subsequent molecular regulatory network.

The performance of the NER, RE, and the integrated automatic literature analysis models was evaluated on a test set.

### Construction and Validation of GENET

The relation triples extracted by the automatic literature analysis model were deduplicated, standardized, and aggregated to create a visual knowledge graph using the AntV G6 framework. In this graph, nodes represent entities, and edges represent the relationships between these entities. To ensure the uniqueness of nodes in GENET, all gene names were integrated from the GENE database within the NCBI database and constructed a table to convert alias names to standard names. The network could be filtered by relation type, relation frequency, publication time, and other parameters (Video S1, Supporting Information).

To evaluate the credibility of molecular relationships across various cancer types, the Pearson correlation coefficient for molecular correlations was calculated using sequencing data from The Cancer Genome Atlas Program (TCGA) database. A random selection of 100 molecular relationships from GENET was used to validate the accuracy of its molecular RE and compare its performance with existing methods.

To demonstrate GENET's potential in uncovering molecular regulatory mechanisms, a case study exploring the regulatory interaction between WNT4 and IGF2 in colon cancer as a case study was conducted. IGF2 and WNT4 were retrieved from the GENET to investigate their interaction. Additionally, bioinformatics techniques were employed to predict the biological functions of the identified molecular pathway in colon cancer. (See Supporting Information). Transcriptome data from 14 patients with colon cancer, along with pathological sections from 25 patients with colon cancer obtained from Renmin Hospital of Wuhan University, were analyzed using multiplex immunofluorescence to validate the identified molecular pathway. The characteristics of the patients involved in the validation are summarized in Table S3 (Supporting Information). The development framework and validation steps of GENET are illustrated in Figure 2. The collection and use of human tissues were approved by the Ethics Committee of Renmin Hospital of Wuhan University.

### Statistical Analysis

The performance of the NER and RE models within the automatic literature analysis system, including GENET, was evaluated using metrics such as precision, recall, and F1 score. Precision is defined as the fraction of true positives out of all observations that are predicted to be positive, calculated as Precision = true positive / (true positive + false positive). Recall is the fraction of true positives detected out of all actual positive labeled examples, calculated as Recall = true positive / (true positive + false negative). The F1 score provides a balance between precision and recall, calculated as F1 = 2 / (1 / recall) + (1 / precision).^[^ ^40^ ^]^

GENET's performance was compared to existing methods using the McNemar test. The expressions of IGF2, WNT4, and identified intermediary regulatory molecules in colon cancer were analyzed using rank‐sum tests and Pearson correlation analysis. A P‐value, which represents the probability that the observed difference could occur under the null hypothesis, was used to assess statistical significance. P < 0.05 was considered statistically significant, indicating a real difference in GENET's performance compared to existing methods.^[^ ^41^ ^]^ All statistical analyses were conducted using SPSS and R software.

## Conflict of Interest

The authors declare no conflict of interest.

## Author Contributions

H.Y., and Q.D. performed conceptualization. J.L., H.Z., J.W., M.D., L.W., Z.D., J.L. performed Data collection; Z.L., J.L., W.J., K.X. performed Algorithm development and implementation. J.L., H.Z., J.W. performed Data analyzing; H.Y., Q.D.performed Project administration; J.L., H.Z., J.W.performed wrote the original draft; H.Y., Q.D., J.L., H.Z., J.W. reviewed and edited the final manuscript.

## Supporting information



Supporting Information

Supplemental Video1

## Data Availability

The data that support the findings of this study are available in the supporting information of this article.
