# Supplementary material for: Development and Validation of an AI‐Driven System for Automatic Literature Analysis and Molecular Regulatory Network Construction
Source: Adv Sci (Weinh). 2024 Oct 7;11(44):2405395. doi: 10.1002/advs.202405395 (PMC11600262; doi:10.1002/advs.202405395)
Supplement: Supplementary file 1 — Supporting Information [file ADVS-11-2405395-s002.pdf]

ADVANCED SCIENCE
Open Access

Supporting Information

for *Adv. Sci.*, DOI 10.1002/advs.202405395

Development and Validation of an AI-Driven System for Automatic Literature Analysis and Molecular Regulatory Network Construction

Jia Li, Hailin Zhang, Jiamin Wang, Mei Deng, Zhiyong Li, Wei Jiang, Kejin Xu, Lianlian Wu, Zehua Dong, Jun Liu, Qianshan Ding and Honggang Yu**

Supporting Information

Development and validation of an AI-driven system for automatic literature analysis and molecular regulatory network construction

Jia Li[1,2,3,4#], Hailin Zhang[1,2,3,4#], Jiamin Wang[1,2,3,4#], Mei Deng[1,2,3,4], Zhiyong Li[4], Wei Jiang[4], Kejin Xu[4], Lianlian Wu[1,2,3,4], Zehua Dong[1,2,3,4], Jun Liu[1,3,5], Qianshan Ding[1,2,3,4], Honggang Yu[1,2,3,4*]*

1. Predictive Details of Molecules Strongly Associated with WNT4 Expression at the Pathological Level

Data Collection and Processing: RNA-seq expression data and pathological slice data were obtained for 456 colon cancer patients from the TCGA database. Exclusion criteria included the absence of slice data (n=16) and slices too small to be sectioned (n=5), resulting in the inclusion of 435 patients' RNA-seq and pathological data. Patients were divided into high and low WNT4 expression groups, yielding 217 patients with low and 218 with high WNT4 expression. Differential gene analysis was conducted using DESeq2, with genes showing significant expression differences between high and low WNT4 expression groups identified by a Padj <0.05 and log2FoldChange >1, totaling 379 genes.

Predictive Model for Molecules Strongly Associated with WNT4 Expression: Pathological slices from 435 patients were uniformly magnified 20 times and cropped to 256×256μm (512×512 pixels) sub-images. Up to 8000 sub-images, each containing over 50% tissue, were randomly selected per slice for model construction. Patients were divided into a training set and a test set at an 8:2 ratio. The molecular prediction model consisted of two parts: feature extraction from image sub-sections and RNA-seq expression prediction. The feature extraction model included a ResNet50 network with a global average pooling layer, producing a one-dimensional output vector of dimensions (2048, 1) from a three-dimensional input vector of (2048, 7, 7). The RNA-seq expression prediction model was a multi-layer neural network that took the extracted feature vector as input with the target value being the RNA-seq expression value (log10 transformed). The training loss function was the mean squared error between predicted and actual RNA-seq expression values. Each image sub-section generated a predictive result for RNA-seq expression, with the aggregate of all sub-sections from a pathological slice providing the final predictive outcome for that image. A five-fold cross-training approach was employed for the molecular prediction model.[1-3] Successful predictions were confirmed using the Pearson correlation coefficient and P-value ($r > 0$ and $P < 0.05$), identifying 33 molecules in total (Table S2). Molecules were prioritized based on correlation strength, molecular type, and volume of literature, with IGF2 selected for further exploration as a molecule strongly associated with WNT4 expression at the pathological level.

2. Details of Functional Enrichment

2.1 Methods

Expression Matrix Acquisition and Preprocessing: The COAD dataset was obtained from the TCGA database, excluding non-cancer samples, resulting in data for 58,387 genes across 469 cancer samples.

Data was normalized using Trimmed Mean of M-values (TMM).[4]

Differential Expression Analysis: The normalized expression matrix was used to segregate samples into high and low expression groups based on median values for specific genes like "WNT4," "STAT3," and "IGF2". Differential expression analysis was then performed using the limma package, with significantly differentially expressed genes (DEGs) identified by an false discovery rate (FDR) ≤ 0.05 and $|\log 2FC| \geq 1$.[5]

Enrichment Analysis: Differential expression results were subjected to enrichment analysis using the clusterProfiler and msigdbr packages, identifying significantly enriched pathways with p-values (adjusted by Benjamini-Hochberg method) ≤ 0.05.

2.2 Results

2.2.1 Differential Expression Analysis

The WNT4 grouping yielded 5,919 DEGs (Up: Down = 686: 5,233), the STAT3 grouping yielded 5,236 DEGs (Up: Down = 530: 4,706), and the IGF2 grouping yielded 3,521 DEGs (Up: Down = 853: 2,668) (Figure S4).

2.2.2 Functional Enrichment Analysis

WNT4, STAT3, and IGF2 enriched 2,338, 4,142, and 11,425 pathways, respectively. The intersection of the three accounted for 340 pathways (Figure S5).

References

1. Y. Wang, K. Kartasalo, P. Weitz, et al., *Cancer Res.* 2021, *81*(19), 5115-5126.
2. P. L. Schrammen, N. Ghaffari Laleh, A. Echle, et al., *J. Pathol.* 2022, *256*(1), 50-60.
3. M. Bilal, S. E. A. Raza, A. Azam, et al., *Lancet Digit. Health* 2021, *3*(12), e763-e772.
4. M. D. Robinson, A. Oshlack, *Genome Biol.* 2010, *11*(3), R25.
5. M. E. Ritchie, B. Phipson, D. Wu, et al., *Nucleic Acids Res.* 2015, *43*(7), e47.

Table S1. Performance of the automatic literature analysis model without biological functions.

	Precision (n/N, 95% CI)	Recall (n/N, 95% CI)	F1 score
**Named entity recognition model of the automatic literature analysis model**			
**Gene**	95.81% (2,586/2,699; 94.99%-96.50%)	98.74% (2,586/2,619; 98.24%-99.10%)	97.25%
**Signal pathway**	95.19% (455/478, 92.89%-96.77%)	98.70% (455/461, 97.19%-99.40%)	96.91%
**Cancer**	95.12% (721/758, 93.35%-96.44%)	98.36% (721/733, 97.16%-99.06%)	96.71%
**Overall**	95.60% (3,762/3,935; 94.91%-96.20%)	98.66% (3,762/3,813; 98.24%-98.98%)	95.72%
**Entity relation extraction model of the automatic literature analysis model**			
**Promotes**	81.01% (495/611, 77.71%-83.92%)	86.84% (495/570, 83.82%-89.37%)	83.82%
**Inhibits**	80.21% (385/480, 76.41%-83.53%)	89.53% (385/430, 86.28%-92.08%)	84.61%
**Upstream**	78.51% (274/349, 73.90%-82.50%)	91.33% (274/300, 87.60%-94.01%)	84.44%
**Abbreviation**	90.63% (319/352, 87.13%-93.25%)	95.80% (319/333, 93.07%-97.48%)	93.14%
**Overall**	82.20% (1,473/1,792; 80.36%-83.90%)	90.20% (1,473/1,633; 88.66%-91.55%)	86.01%
**Entity relation extraction model of integrated automatic literature analysis model**			
**Promotes**	87.90% (494/562, 84.94%-90.34%)	86.67% (494/570, 83.63%-89.22%)	87.28%
**Inhibits**	85.81% (375/437, 82.23%-88.77%)	87.21% (375/430, 83.72%-90.04%)	86.50%
**Upstream**	84.69% (271/320, 80.34%-88.22%)	90.33% (271/300, 86.46%-93.18%)	87.42%
**Abbreviation**	94.03% (315/335, 90.96%-96.10%)	94.59% (315/333, 91.61%-96.55%)	94.31%
**Overall**	87.97% (1,455/1,654; 86.31%-89.45%)	89.10% (1,455/1,633; 87.50%-90.52%)	88.53%

Table S2. Performance of molecular prediction models strongly correlated with WNT4 expression at the pathological level.

Ensembl ID	Gene	r	p-value
ENSG00000182256	GABRG3	0.63	＜0.001
ENSG00000196758	AC079612.1	0.44	＜0.001
ENSG00000244743	AC087588.1	0.36	＜0.001
ENSG00000167244	IGF2	0.35	＜0.001
ENSG00000278505	C17orf78	0.35	＜0.001
ENSG00000238133	MAP3K20-AS1	0.33	＜0.01
ENSG00000224555	AC087441.2	0.32	＜0.01
ENSG00000275772	AC244157.2	0.30	＜0.01
ENSG00000007402	CACNA2D2	0.28	＜0.01
ENSG00000240563	L1TD1	0.28	＜0.01
ENSG00000203690	TCP10	0.28	＜0.01
ENSG00000204019	CT83	0.26	0.01
ENSG00000249853	HS3ST5	0.26	0.01
ENSG00000197893	NRAP	0.26	0.01
ENSG00000104327	CALB1	0.25	0.02
ENSG00000148677	ANKRD1	0.25	0.02
ENSG00000261780	LINC02582	0.24	0.02
ENSG00000154529	CNTNAP3B	0.24	0.02
ENSG00000005981	ASB4	0.24	0.02
ENSG00000225972	MTND1P23	0.23	0.03
ENSG00000260337	AC091544.4	0.23	0.03
ENSG00000173612	GPRC6A	0.23	0.03
ENSG00000007216	SLC13A2	0.23	0.03
ENSG00000123561	SERPINA7	0.23	0.03
ENSG00000178343	SHISA3	0.22	0.04
ENSG00000172000	ZNF556	0.22	0.04
ENSG00000165973	NELL1	0.22	0.04
ENSG00000187472	AL589826.1	0.22	0.04
ENSG00000088726	TMEM40	0.22	0.04
ENSG00000233214	AC002511.2	0.21	0.04
ENSG00000145198	VWA5B2	0.21	0.04
ENSG00000261226	AC092384.3	0.21	0.04
ENSG00000161798	AQP5	0.21	0.05

Table S3. Characteristics of the patients used for validation.

Characteristics	Transcriptome (n=14)	Multiplex immunofluorescence (n=25)
**Age, years (SD)**	63.71 (14.68)	67.60 (8.55)
**Sex, n (%)**		
**Female**	4 (28.57)	13 (52.00)
**Male**	10 (71.43)	12 (48.00)
**Pathology, n (%)**		
**poorly-differentiated adenocarcinoma**	0	3 (12.00)
**moderately-differentiated adenocarcinoma**	14 (100)	21 (84.00)
**well-differentiated adenocarcinoma**	0	1 (4.00)
**T-staging, n (%)**		
**Tis**	0	0
**T1**	0	1 (4.00)
**T2**	5 (35.71)	4 (16.00)
**T3**	0	3 (12.00)
**T4a**	9 (64.29)	17 (68.00)
**T4b**	0	0
**Intravascular tumor thrombus, n (%)**	3 (21.43)	11 (44.0)
**Perineural invasion, n (%)**	7 (50.00)	8 (32.00)
**Lymph node metastasis of carcinomas, n (%)**	3 (21.43)	10 (40.00)

SD: standard deviation.

Figure legends

Figure S1. Confusion Matrix of the automatic literature analysis model. (A) Confusion Matrix of named entity recognition model of automatic literature analysis model. (B) Confusion Matrix of entity relation extraction model of automatic literature analysis model. (C) Confusion Matrix of entity relation extraction model of integrated automatic literature analysis model.

Figure S2. Confusion Matrix of the automatic literature analysis model without biological functions. (A) Confusion Matrix of named entity recognition model of automatic literature analysis model without biological functions. (B) Confusion Matrix of entity relation extraction model of automatic literature analysis model without biological functions. (C) Confusion Matrix of entity relation extraction model of integrated automatic literature analysismodel without biological functions.

Figure S3. Performance of GENET in molecular relation extraction compared to existing databases. (A) Performance of GENET in molecular relation extraction. (B) Performance of BioGRID in molecular relation extraction. (C) Performance of GeneMANIA in molecular relation extraction. (D) Performance of GEPI in molecular relation extraction. (E) Performance of STRING in molecular relation extraction. (F) Performance of GENIE3 in molecular relation extraction. (G) Performance of GRNBoost2 in molecular relation extraction. (H) Performance of KBoost in molecular relation extraction. (I) Performance of STGRNS in molecular relation extraction.

Figure S4. Results of the differential expression and functional enrichment analyses. (A) Results of the differential expression analysis based on the WNT4 expression level. (B) Results of the differential expression analysis based on STAT3 expression level. (C) A Venn diagram of functional enrichment.

Figure S5. Results of 340 functional enrichment analyses.

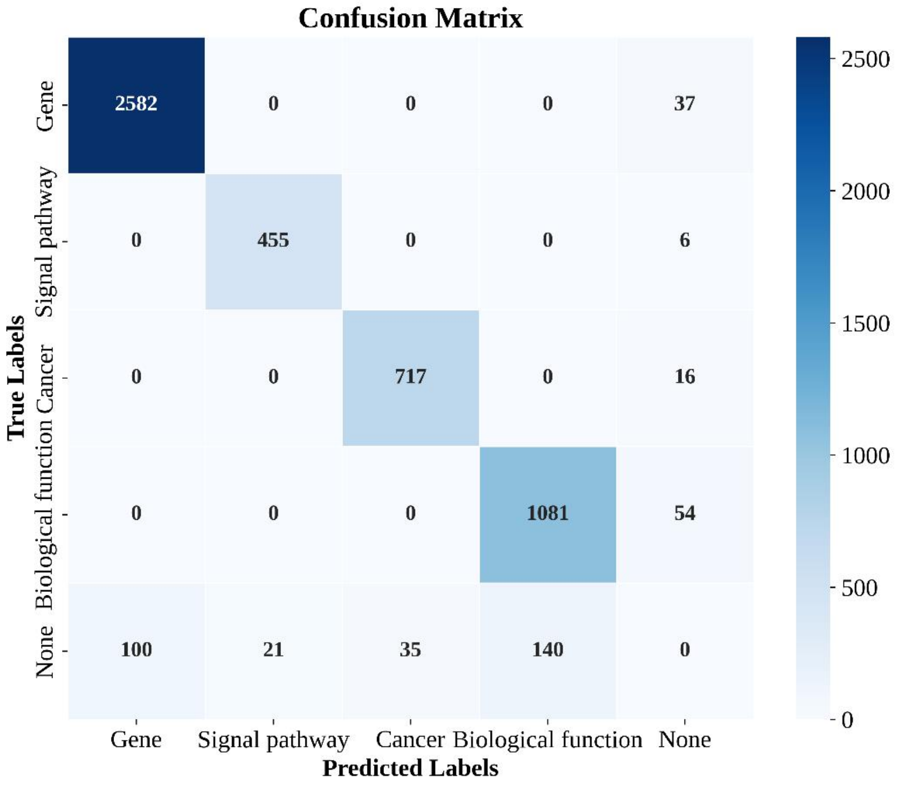

Figure S1A. Confusion Matrix of named entity recognition model of automatic literature analysis model.

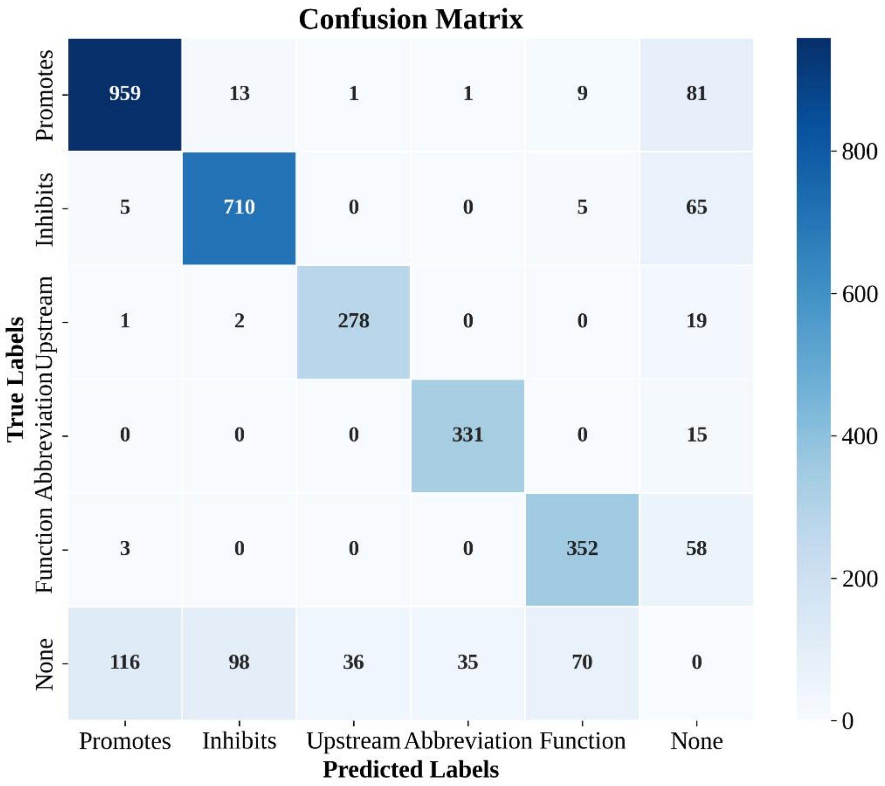

Figure S1B. Confusion Matrix of entity relation extraction model of automatic literature analysis model.

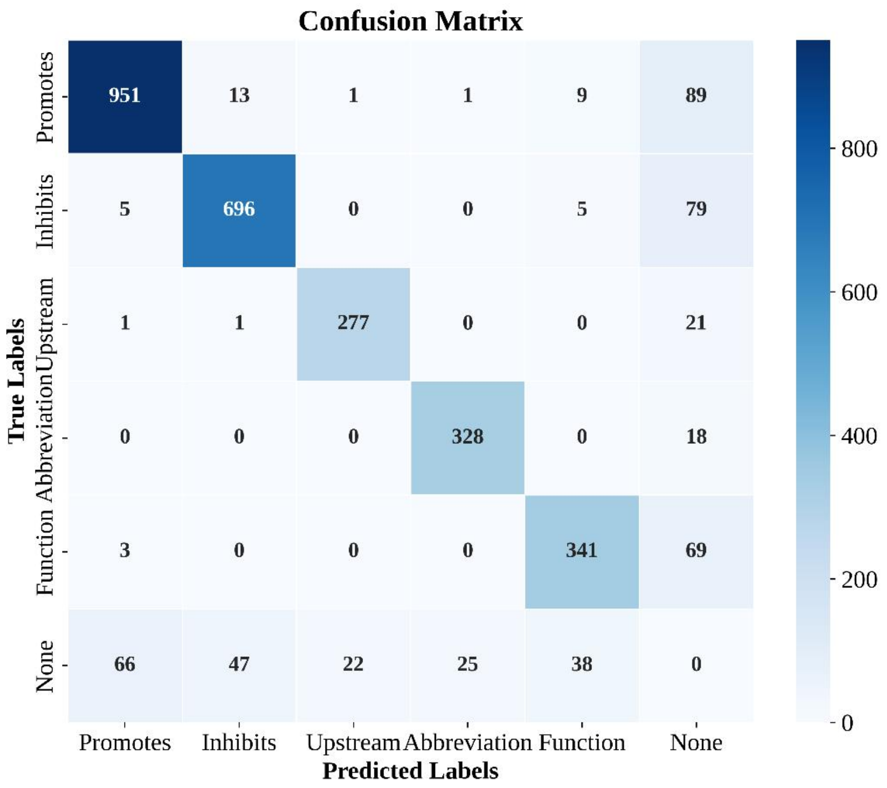

Figure S1C. Confusion Matrix of entity relation extraction model of integrated automatic literature analysis model.

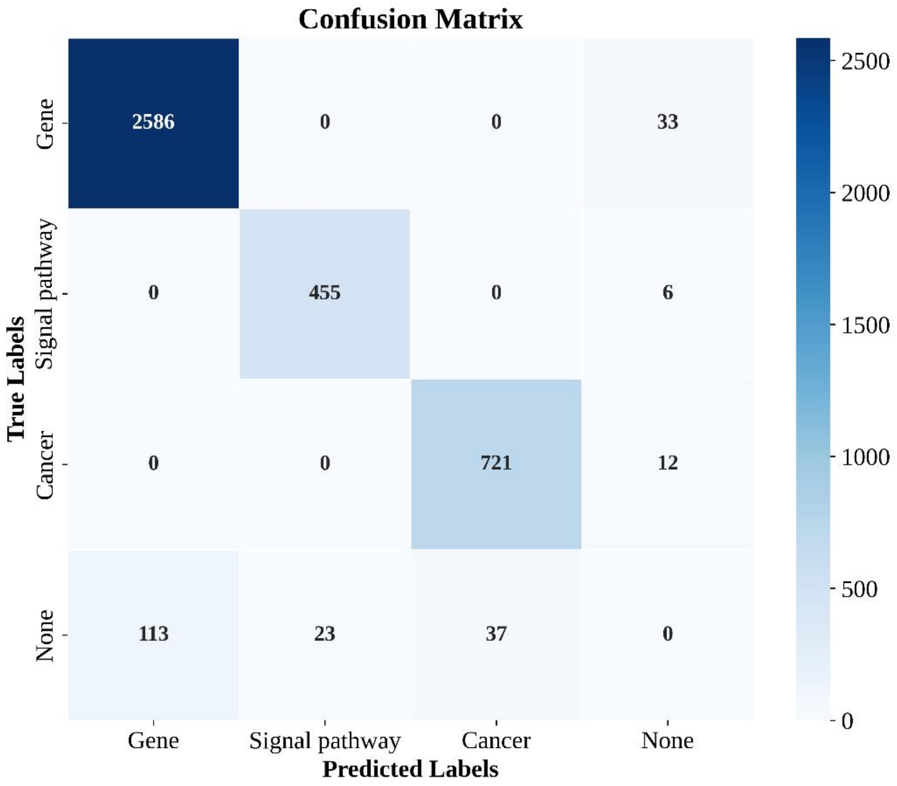

Figure S2A. Confusion Matrix of named entity recognition model of automatic literature analysis model without biological functions.

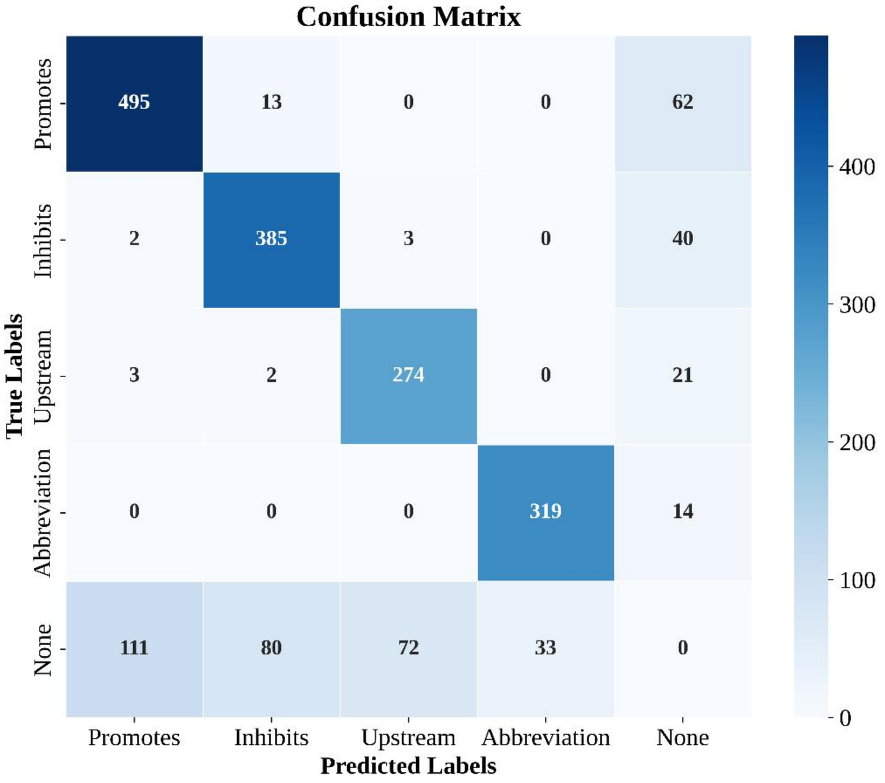

Figure S2B. Confusion Matrix of entity relation extraction model of automatic literature analysis model without biological functions.

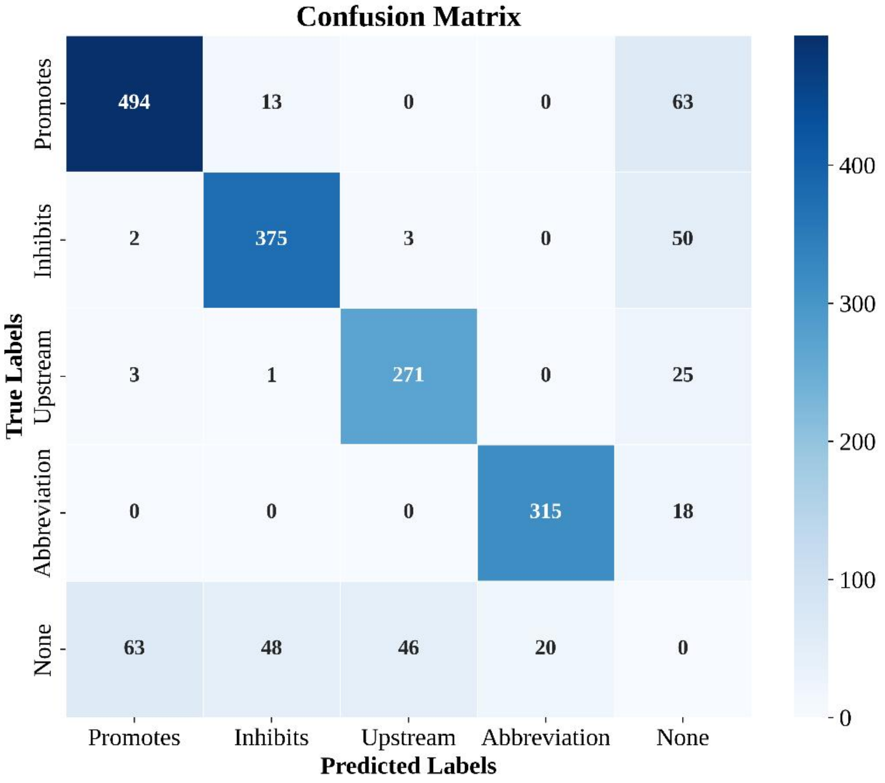

Figure S2C. Confusion Matrix of entity relation extraction model of integrated automatic literature analysis model without biological functions.

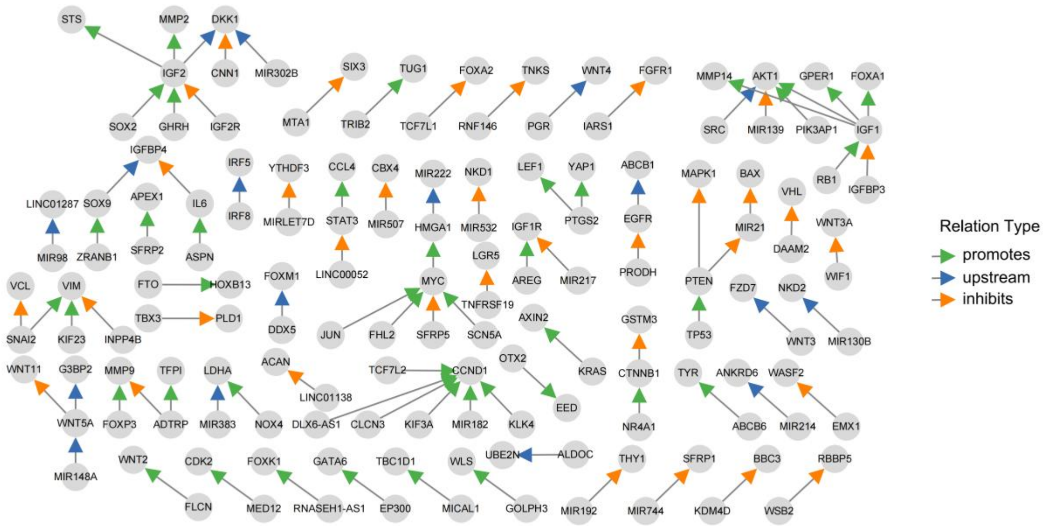

Figure S3A. Performance of GENET in molecular relation extraction.

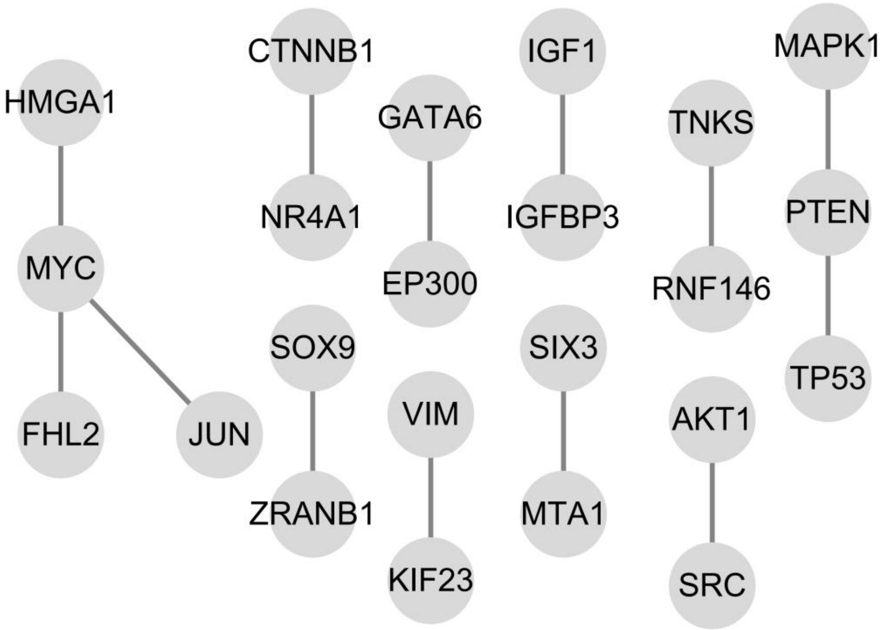

Figure S3B. Performance of BioGRID in molecular relation extraction.

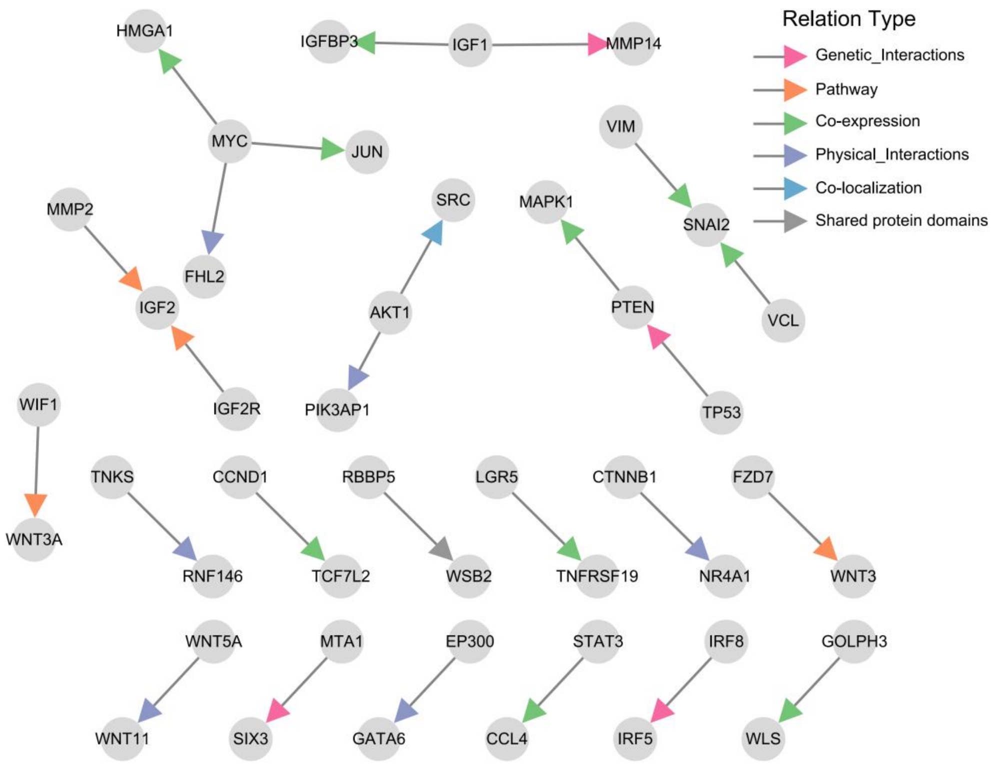

Figure S3C. Performance of GeneMANIA in molecular relation extraction.

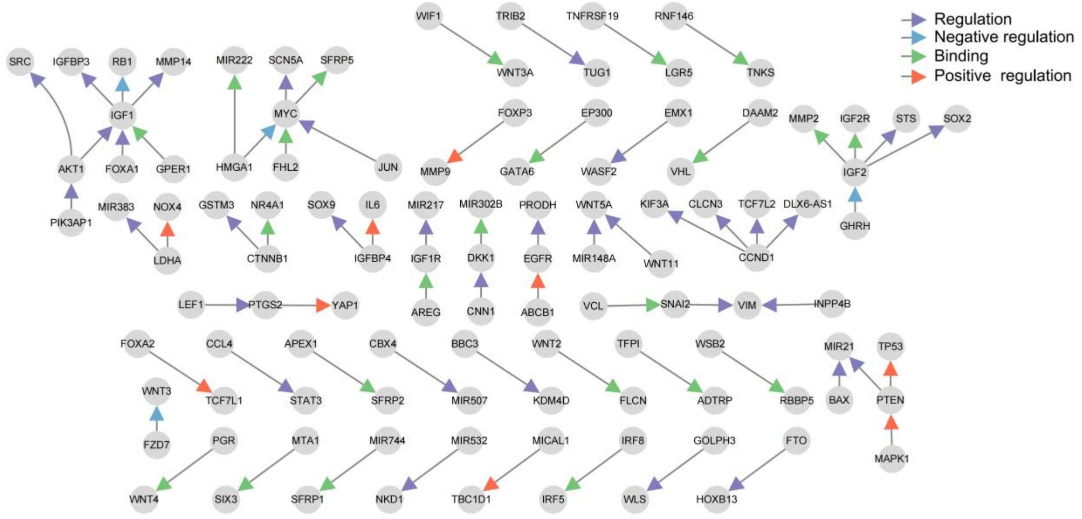

Figure S3D. Performance of GEPI in molecular relation extraction.

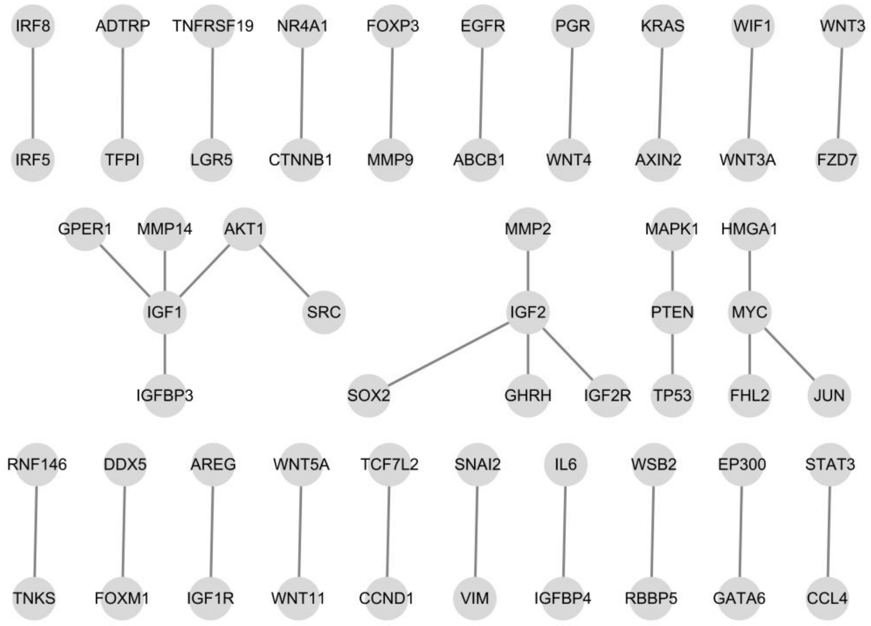

Figure S3E. Performance of STRING in molecular relation extraction.

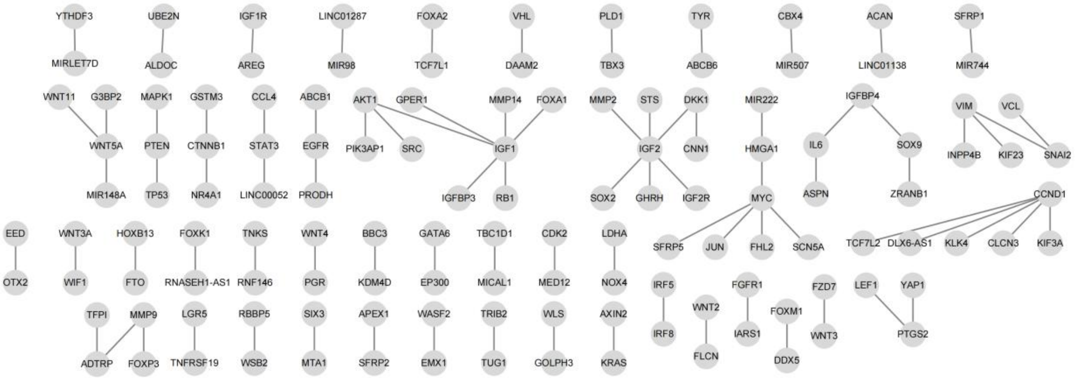

Figure S3F. Performance of GENIE3 in molecular relation extraction.

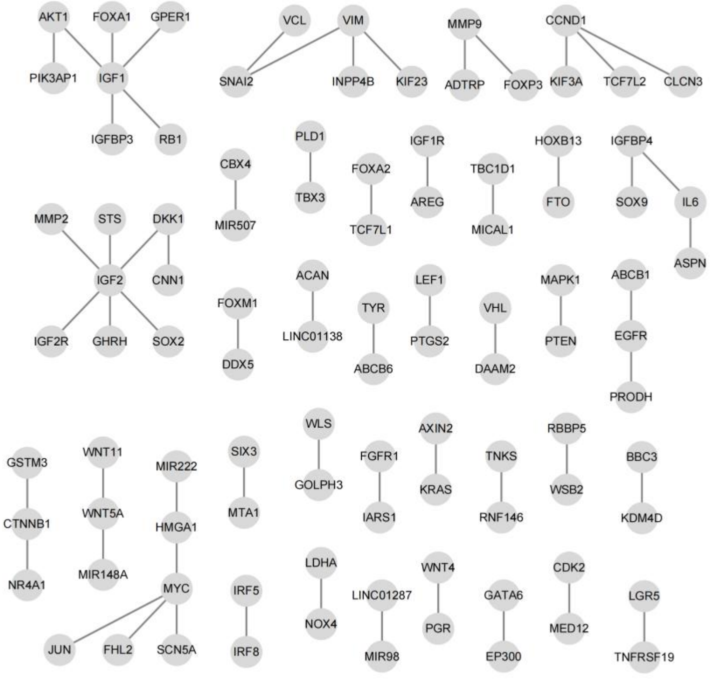

Figure S3G. Performance of GRNBoost2 in molecular relation extraction.

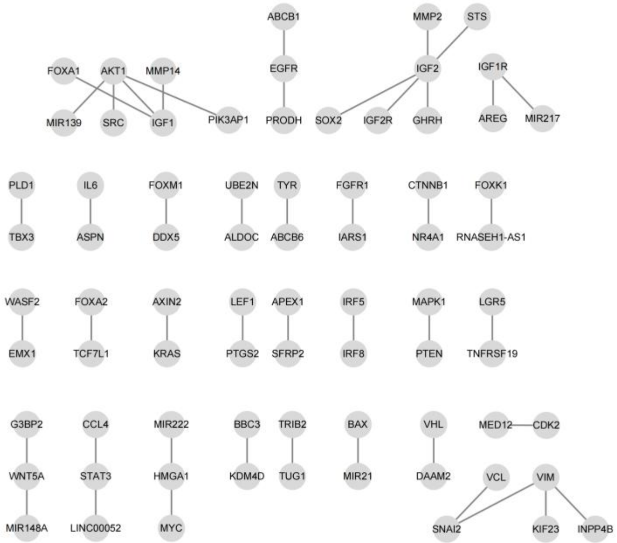

Figure S3H. Performance of KBoost in molecular relation extraction.

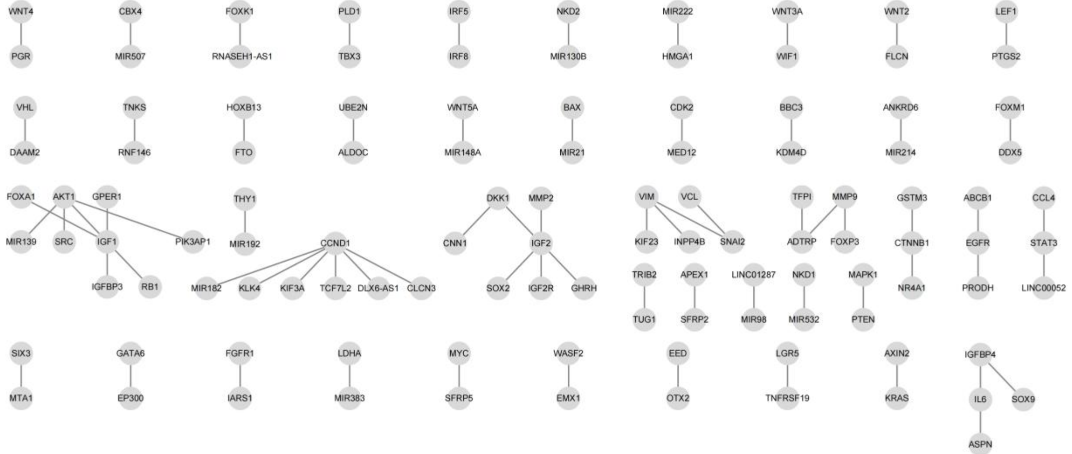

Figure S3I. Performance of STGRNS in molecular relation extraction.

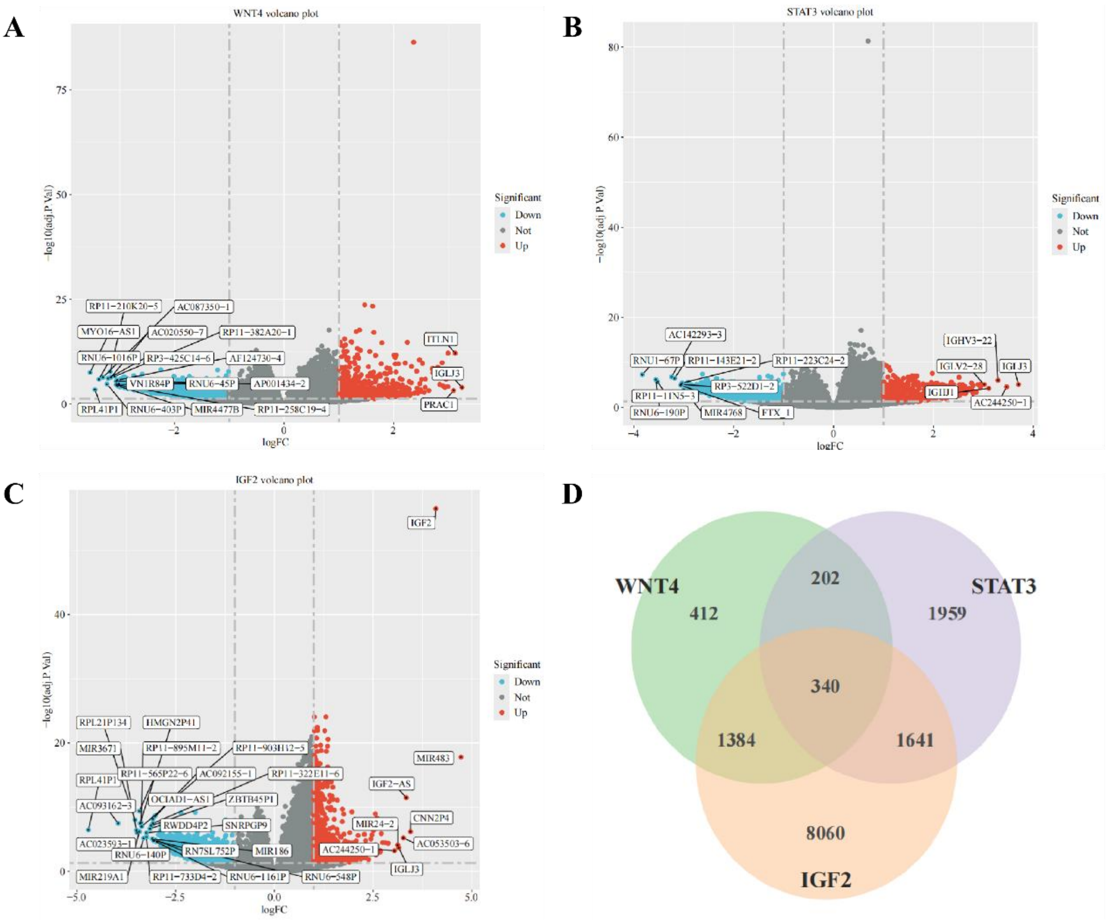

Figure S4. Results of the differential expression and functional enrichment analyses. (A) Results of the differential expression analysis based on WNT4 expression level. (B) Results of the differential expression analysis based on STAT3 expression level. (C) A Venn diagram of functional enrichment.

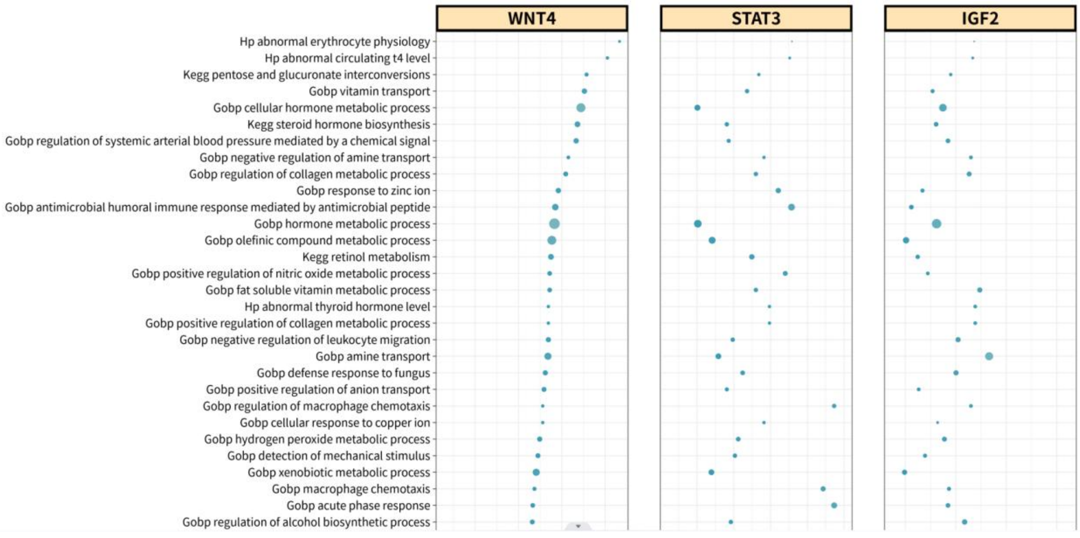

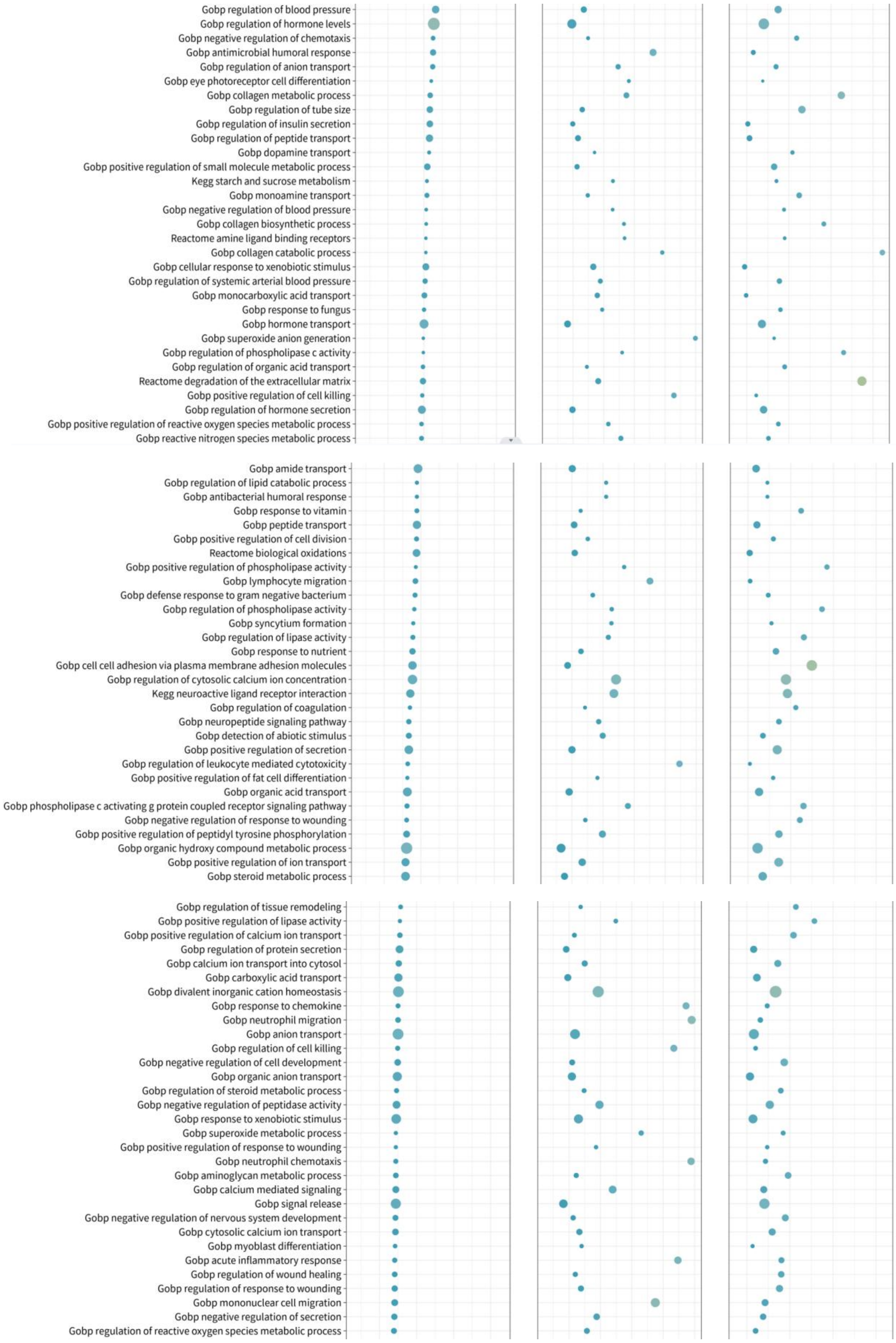
Gobp regulation of blood pressure
Gobp regulation of hormone levels
Gobp negative regulation of chemotaxis
Gobp antimicrobial humoral response
Gobp regulation of anion transport
Gobp eye photoreceptor cell differentiation
Gobp collagen metabolic process
Gobp regulation of tube size
Gobp regulation of insulin secretion
Gobp regulation of peptide transport
Gobp dopamine transport
Gobp positive regulation of small molecule metabolic process
Kegg starch and sucrose metabolism
Gobp monoamine transport
Gobp negative regulation of blood pressure
Gobp collagen biosynthetic process
Reactome amine ligand binding receptors
Gobp collagen catabolic process
Gobp cellular response to xenobiotic stimulus
Gobp regulation of systemic arterial blood pressure
Gobp monocarboxylic acid transport
Gobp response to fungus
Gobp hormone transport
Gobp superoxide anion generation
Gobp regulation of phospholipase c activity
Gobp regulation of organic acid transport
Reactome degradation of the extracellular matrix
Gobp positive regulation of cell killing
Gobp regulation of hormone secretion
Gobp positive regulation of reactive oxygen species metabolic process
Gobp reactive nitrogen species metabolic process
Gobp amide transport
Gobp regulation of lipid catabolic process
Gobp antibacterial humoral response
Gobp response to vitamin
Gobp peptide transport
Gobp positive regulation of cell division
Reactome biological oxidations
Gobp positive regulation of phospholipase activity
Gobp lymphocyte migration
Gobp defense response to gram negative bacterium
Gobp regulation of phospholipase activity
Gobp syncytium formation
Gobp regulation of lipase activity
Gobp response to nutrient
Gobp cell cell adhesion via plasma membrane adhesion molecules
Gobp regulation of cytosolic calcium ion concentration
Kegg neuroactive ligand receptor interaction
Gobp regulation of coagulation
Gobp neuropeptide signaling pathway
Gobp detection of abiotic stimulus
Gobp positive regulation of secretion
Gobp regulation of leukocyte mediated cytotoxicity
Gobp positive regulation of fat cell differentiation
Gobp organic acid transport
Gobp phospholipase c activating g protein coupled receptor signaling pathway
Gobp negative regulation of response to wounding
Gobp positive regulation of peptidyl tyrosine phosphorylation
Gobp organic hydroxy compound metabolic process
Gobp positive regulation of ion transport
Gobp steroid metabolic process
Gobp regulation of tissue remodeling
Gobp positive regulation of lipase activity
Gobp positive regulation of calcium ion transport
Gobp regulation of protein secretion
Gobp calcium ion transport into cytosol
Gobp carboxylic acid transport
Gobp divalent inorganic cation homeostasis
Gobp response to chemokine
Gobp neutrophil migration
Gobp anion transport
Gobp regulation of cell killing
Gobp negative regulation of cell development
Gobp organic anion transport
Gobp regulation of steroid metabolic process
Gobp negative regulation of peptidase activity
Gobp response to xenobiotic stimulus
Gobp superoxide metabolic process
Gobp positive regulation of response to wounding
Gobp neutrophil chemotaxis
Gobp aminoglycan metabolic process
Gobp calcium mediated signaling
Gobp signal release
Gobp negative regulation of nervous system development
Gobp cytosolic calcium ion transport
Gobp myoblast differentiation
Gobp acute inflammatory response
Gobp regulation of wound healing
Gobp regulation of response to wounding
Gobp mononuclear cell migration
Gobp negative regulation of secretion
Gobp regulation of reactive oxygen species metabolic process

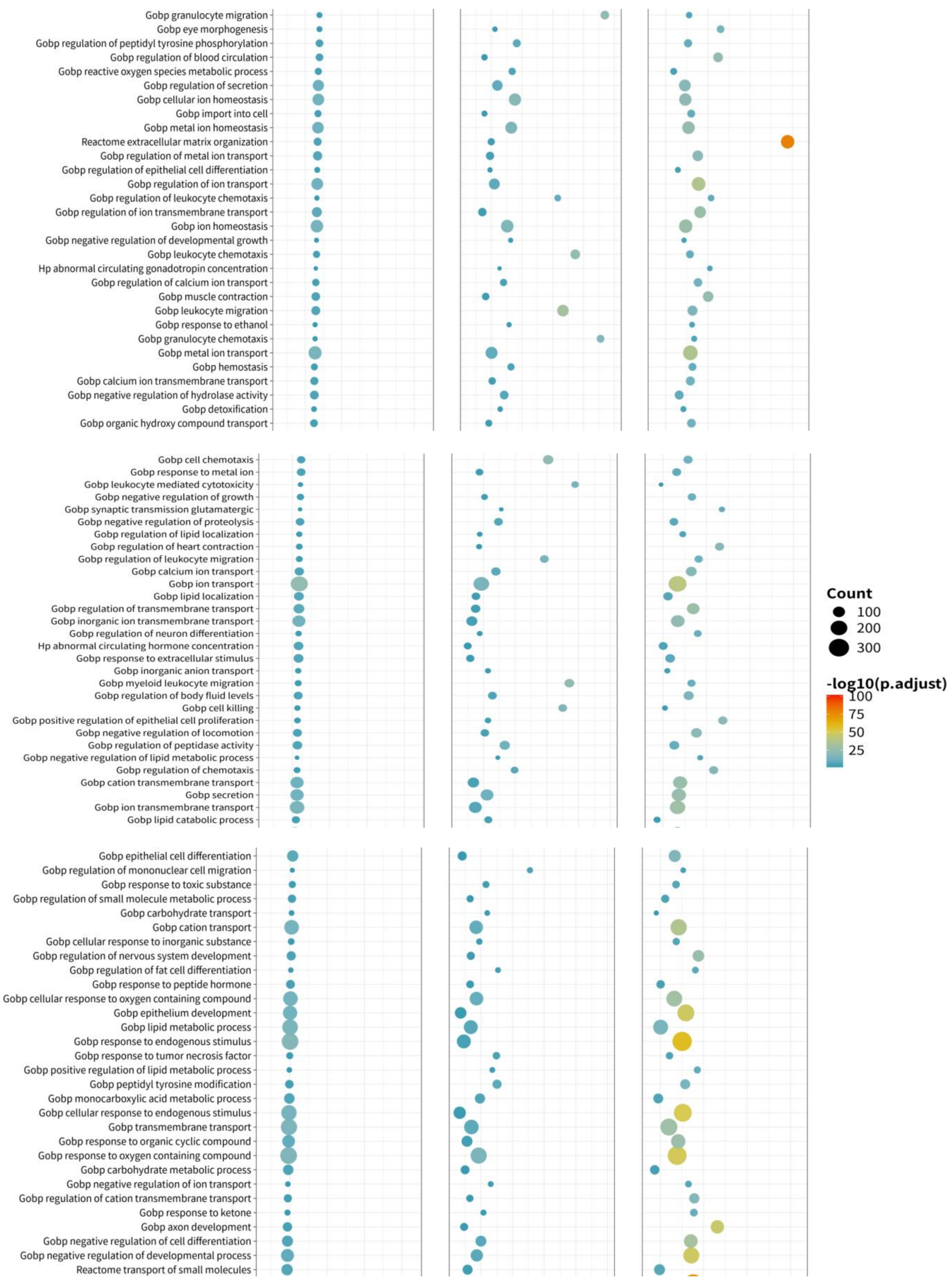
Gobp granulocyte migration
Gobp eye morphogenesis
Gobp regulation of peptidyl tyrosine phosphorylation
Gobp regulation of blood circulation
Gobp reactive oxygen species metabolic process
Gobp regulation of secretion
Gobp cellular ion homeostasis
Gobp import into cell
Gobp metal ion homeostasis
Reactome extracellular matrix organization
Gobp regulation of metal ion transport
Gobp regulation of epithelial cell differentiation
Gobp regulation of ion transport
Gobp regulation of leukocyte chemotaxis
Gobp regulation of ion transmembrane transport
Gobp ion homeostasis
Gobp negative regulation of developmental growth
Gobp leukocyte chemotaxis
Hp abnormal circulating gonadotropin concentration
Gobp regulation of calcium ion transport
Gobp muscle contraction
Gobp leukocyte migration
Gobp response to ethanol
Gobp granulocyte chemotaxis
Gobp metal ion transport
Gobp hemostasis
Gobp calcium ion transmembrane transport
Gobp negative regulation of hydrolase activity
Gobp detoxification
Gobp organic hydroxy compound transport
Gobp cell chemotaxis
Gobp response to metal ion
Gobp leukocyte mediated cytotoxicity
Gobp negative regulation of growth
Gobp synaptic transmission glutamatergic
Gobp negative regulation of proteolysis
Gobp regulation of lipid localization
Gobp regulation of heart contraction
Gobp regulation of leukocyte migration
Gobp calcium ion transport
Gobp ion transport
Gobp lipid localization
Gobp regulation of transmembrane transport
Gobp inorganic ion transmembrane transport
Gobp regulation of neuron differentiation
Hp abnormal circulating hormone concentration
Gobp response to extracellular stimulus
Gobp inorganic anion transport
Gobp myeloid leukocyte migration
Gobp regulation of body fluid levels
Gobp cell killing
Gobp positive regulation of epithelial cell proliferation
Gobp negative regulation of locomotion
Gobp regulation of peptidase activity
Gobp negative regulation of lipid metabolic process
Gobp regulation of chemotaxis
Gobp cation transmembrane transport
Gobp secretion
Gobp ion transmembrane transport
Gobp lipid catabolic process
Gobp epithelial cell differentiation
Gobp regulation of mononuclear cell migration
Gobp response to toxic substance
Gobp regulation of small molecule metabolic process
Gobp carbohydrate transport
Gobp cation transport
Gobp cellular response to inorganic substance
Gobp regulation of nervous system development
Gobp regulation of fat cell differentiation
Gobp response to peptide hormone
Gobp cellular response to oxygen containing compound
Gobp epithelium development
Gobp lipid metabolic process
Gobp response to endogenous stimulus
Gobp response to tumor necrosis factor
Gobp positive regulation of lipid metabolic process
Gobp peptidyl tyrosine modification
Gobp monocarboxylic acid metabolic process
Gobp cellular response to endogenous stimulus
Gobp transmembrane transport
Gobp response to organic cyclic compound
Gobp response to oxygen containing compound
Gobp carbohydrate metabolic process
Gobp negative regulation of ion transport
Gobp regulation of cation transmembrane transport
Gobp response to ketone
Gobp axon development
Gobp negative regulation of cell differentiation
Gobp negative regulation of developmental process
Reactome transport of small molecules
Count
100
200
300
-log10(p.adjust)
100
75
50
25

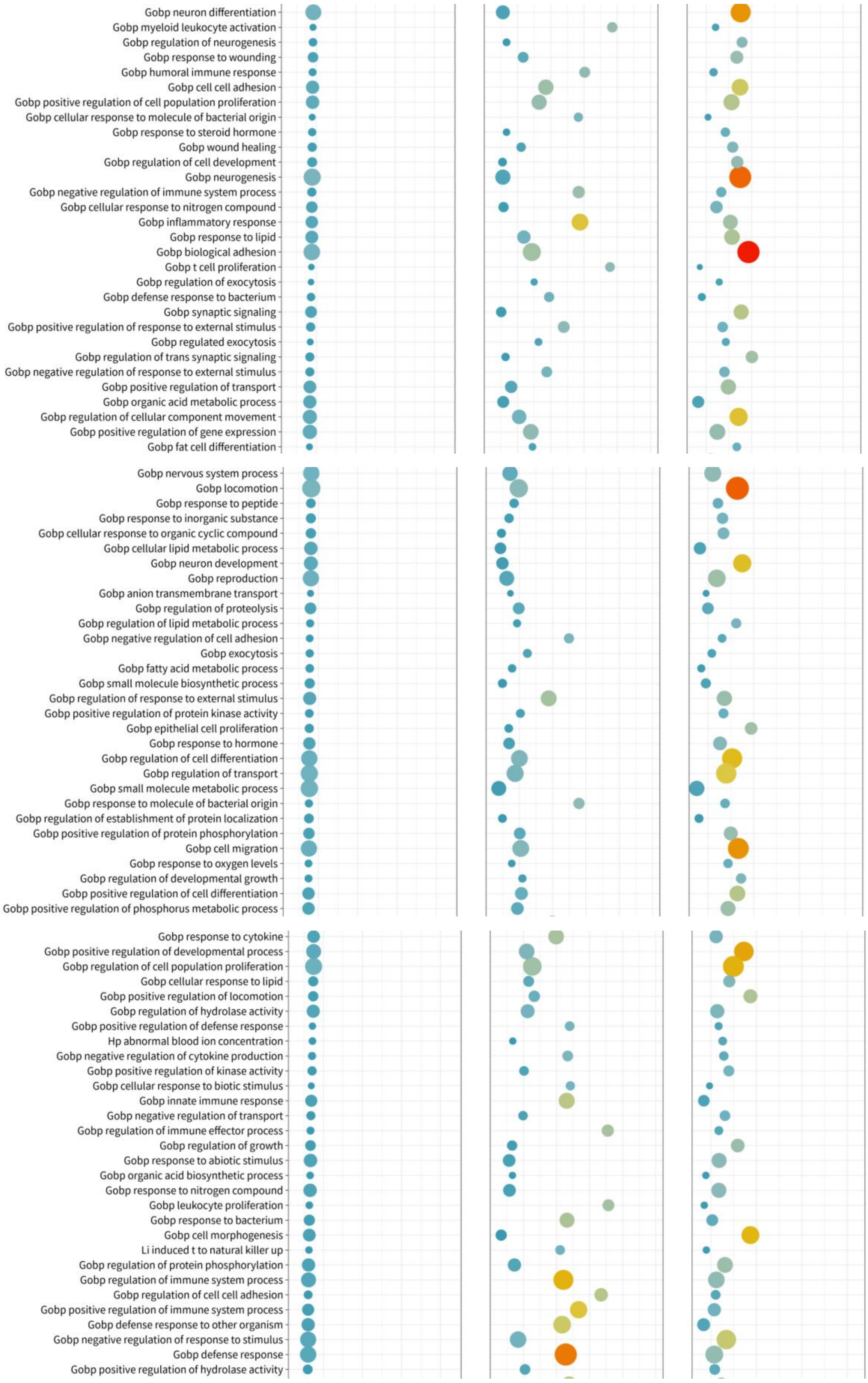
Gobp neuron differentiation
Gobp myeloid leukocyte activation
Gobp regulation of neurogenesis
Gobp response to wounding
Gobp humoral immune response
Gobp cell cell adhesion
Gobp positive regulation of cell population proliferation
Gobp cellular response to molecule of bacterial origin
Gobp response to steroid hormone
Gobp wound healing
Gobp regulation of cell development
Gobp neurogenesis
Gobp negative regulation of immune system process
Gobp cellular response to nitrogen compound
Gobp inflammatory response
Gobp response to lipid
Gobp biological adhesion
Gobp t cell proliferation
Gobp regulation of exocytosis
Gobp defense response to bacterium
Gobp synaptic signaling
Gobp positive regulation of response to external stimulus
Gobp regulated exocytosis
Gobp regulation of trans synaptic signaling
Gobp negative regulation of response to external stimulus
Gobp positive regulation of transport
Gobp organic acid metabolic process
Gobp regulation of cellular component movement
Gobp positive regulation of gene expression
Gobp fat cell differentiation
Gobp nervous system process
Gobp locomotion
Gobp response to peptide
Gobp response to inorganic substance
Gobp cellular response to organic cyclic compound
Gobp cellular lipid metabolic process
Gobp neuron development
Gobp reproduction
Gobp anion transmembrane transport
Gobp regulation of proteolysis
Gobp regulation of lipid metabolic process
Gobp negative regulation of cell adhesion
Gobp exocytosis
Gobp fatty acid metabolic process
Gobp small molecule biosynthetic process
Gobp regulation of response to external stimulus
Gobp positive regulation of protein kinase activity
Gobp epithelial cell proliferation
Gobp response to hormone
Gobp regulation of cell differentiation
Gobp regulation of transport
Gobp small molecule metabolic process
Gobp response to molecule of bacterial origin
Gobp regulation of establishment of protein localization
Gobp positive regulation of protein phosphorylation
Gobp cell migration
Gobp response to oxygen levels
Gobp regulation of developmental growth
Gobp positive regulation of cell differentiation
Gobp positive regulation of phosphorus metabolic process
Gobp response to cytokine
Gobp positive regulation of developmental process
Gobp regulation of cell population proliferation
Gobp cellular response to lipid
Gobp positive regulation of locomotion
Gobp regulation of hydrolase activity
Gobp positive regulation of defense response
Hp abnormal blood ion concentration
Gobp negative regulation of cytokine production
Gobp positive regulation of kinase activity
Gobp cellular response to biotic stimulus
Gobp innate immune response
Gobp negative regulation of transport
Gobp regulation of immune effector process
Gobp regulation of growth
Gobp response to abiotic stimulus
Gobp organic acid biosynthetic process
Gobp response to nitrogen compound
Gobp leukocyte proliferation
Gobp response to bacterium
Gobp cell morphogenesis
Li induced t to natural killer up
Gobp regulation of protein phosphorylation
Gobp regulation of immune system process
Gobp regulation of cell cell adhesion
Gobp positive regulation of immune system process
Gobp defense response to other organism
Gobp negative regulation of response to stimulus
Gobp defense response
Gobp positive regulation of hydrolase activity

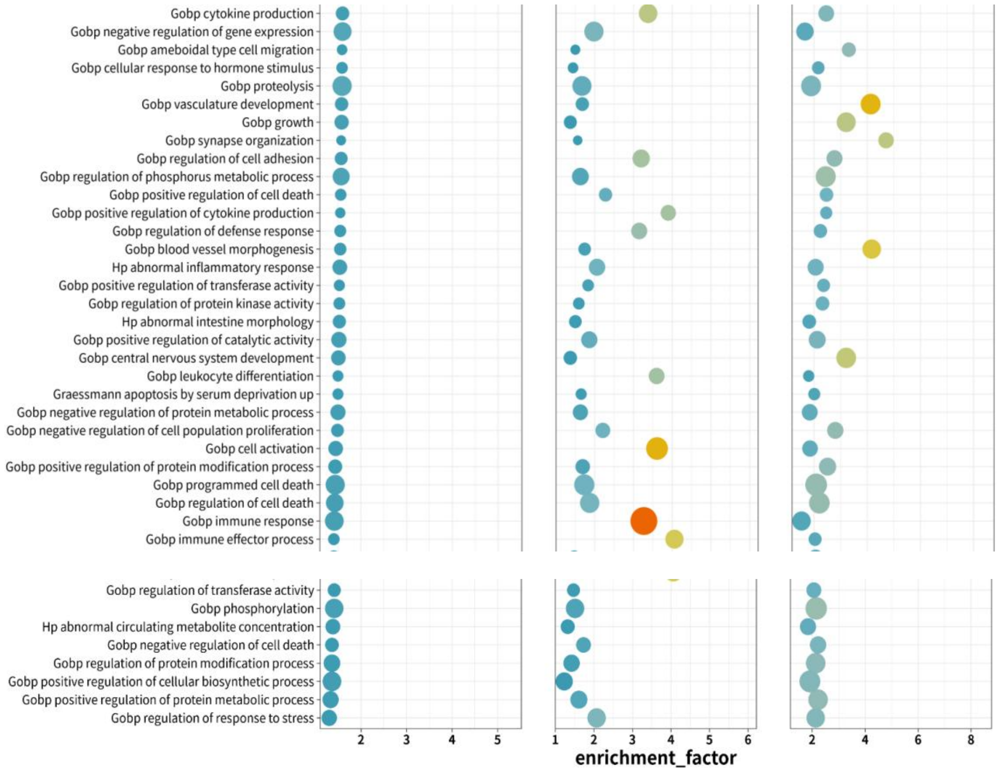

Figure S5. Results of 340 functional enrichment analyses.

Results of multiplex immunofluorescence

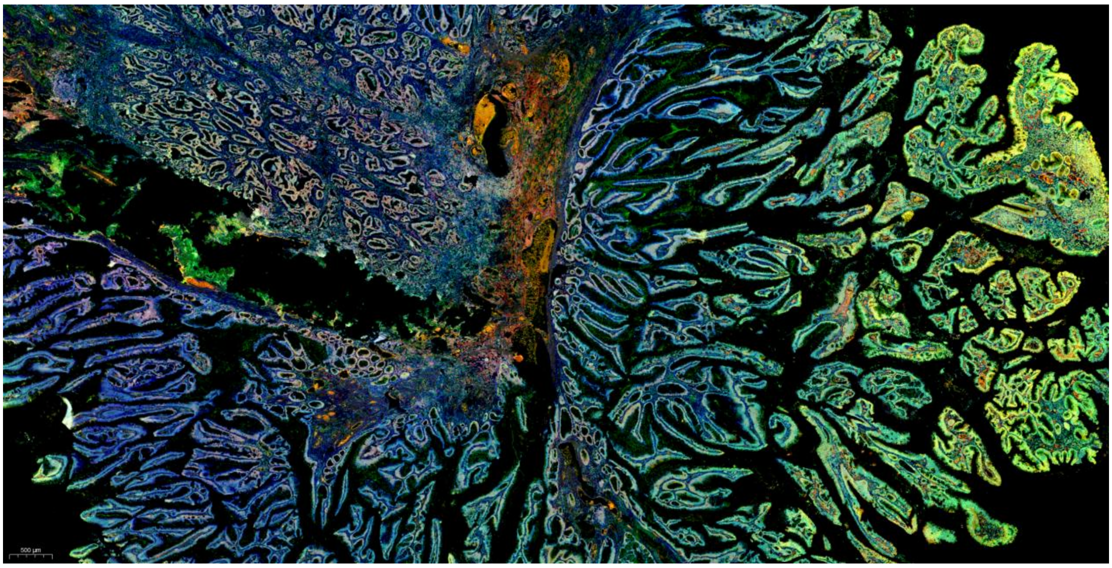

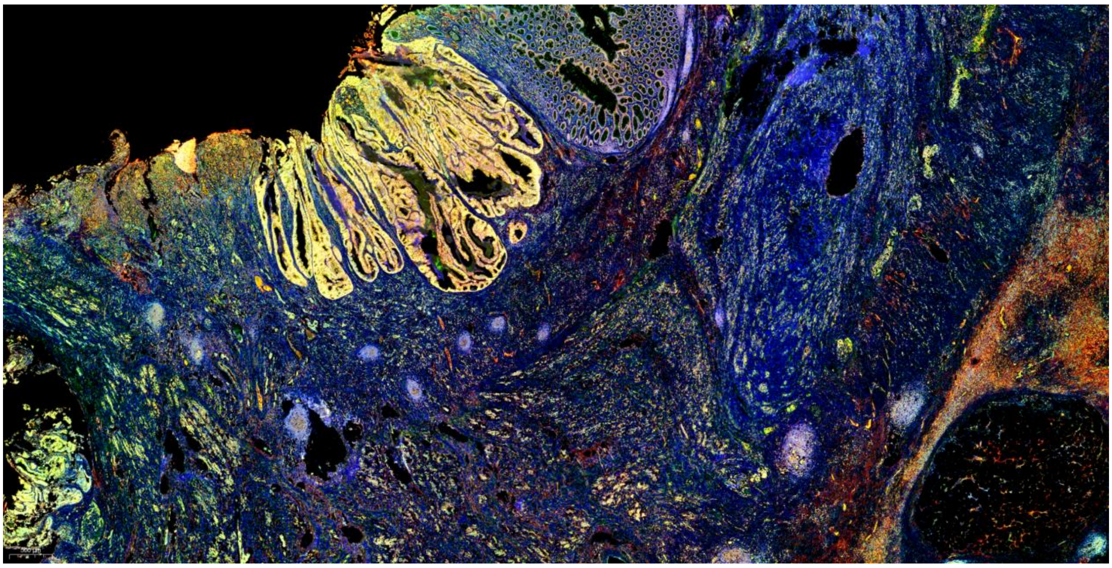

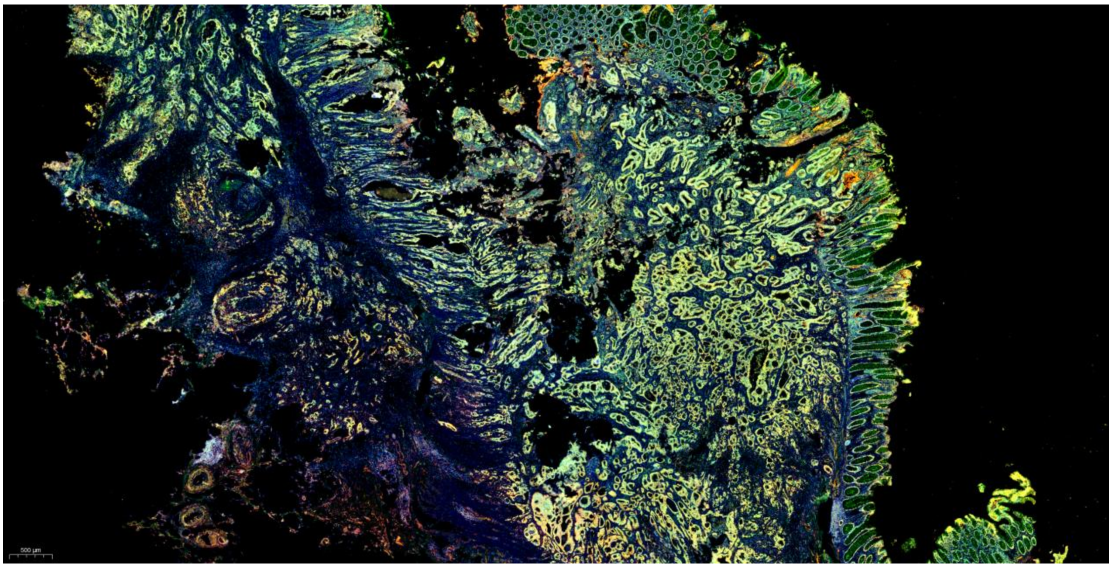

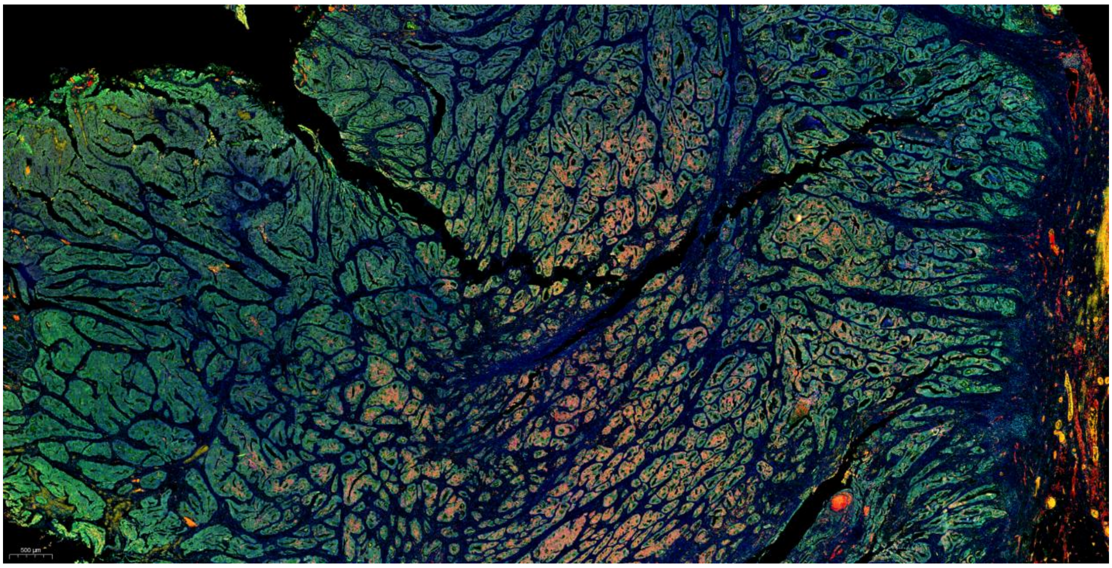
500 µm

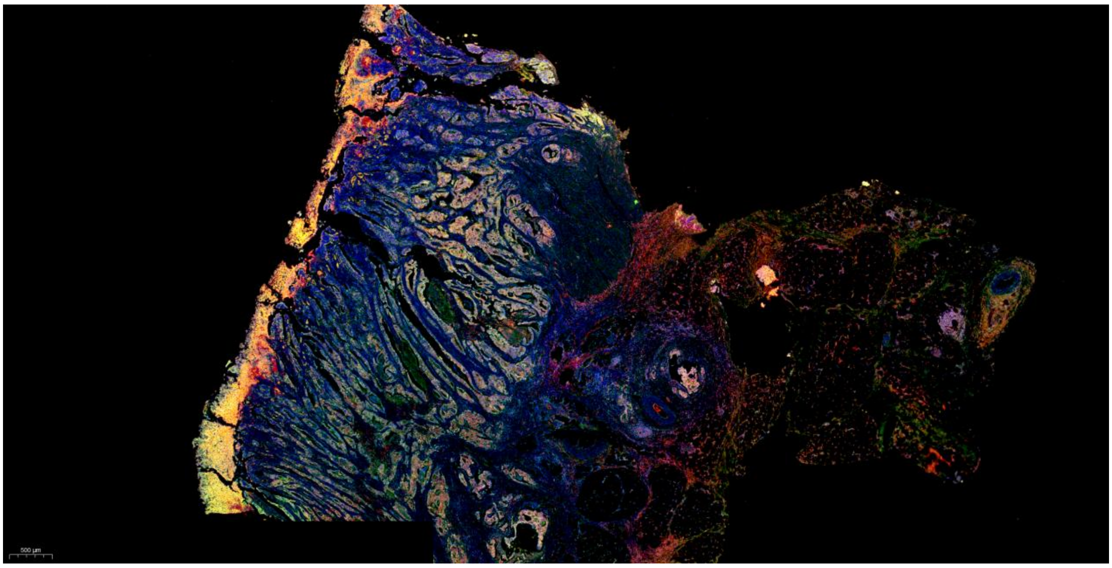
500 µm

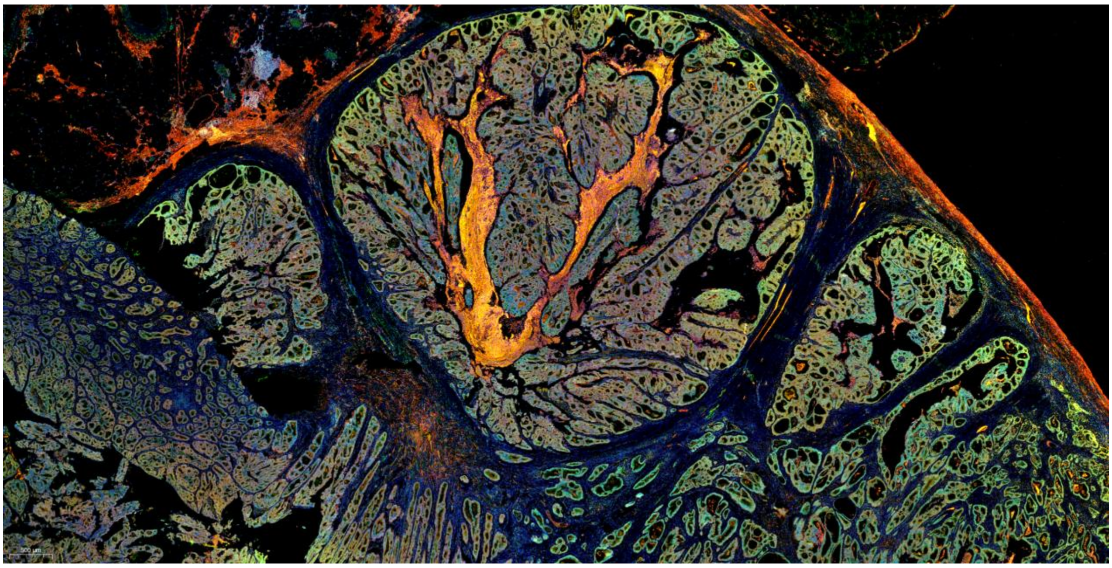
500 µm

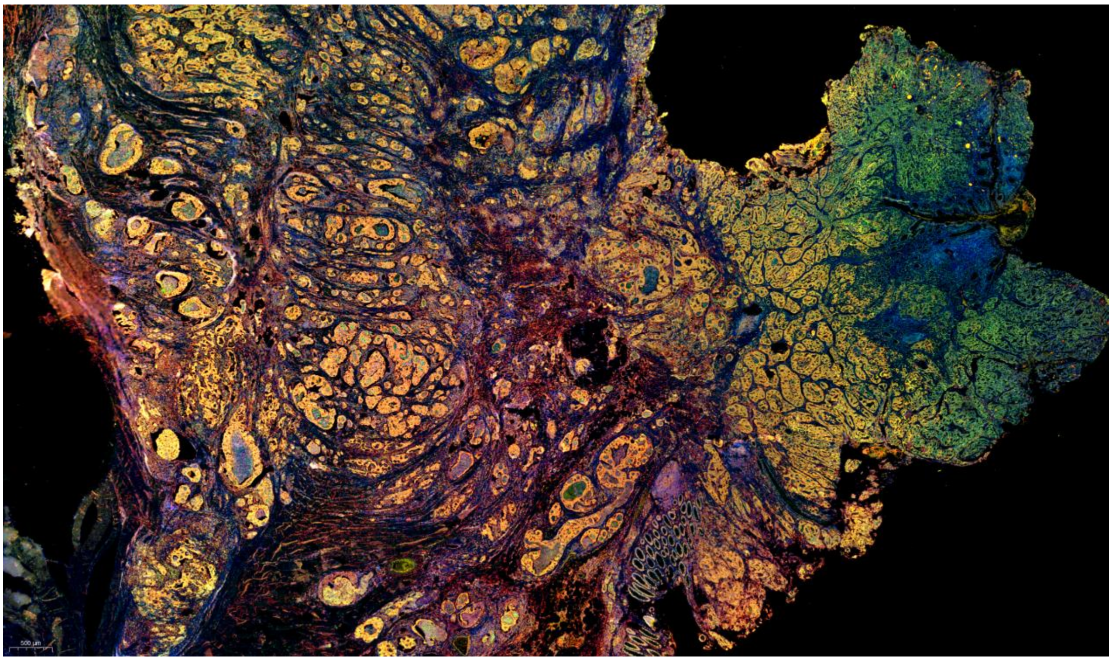
500 µm

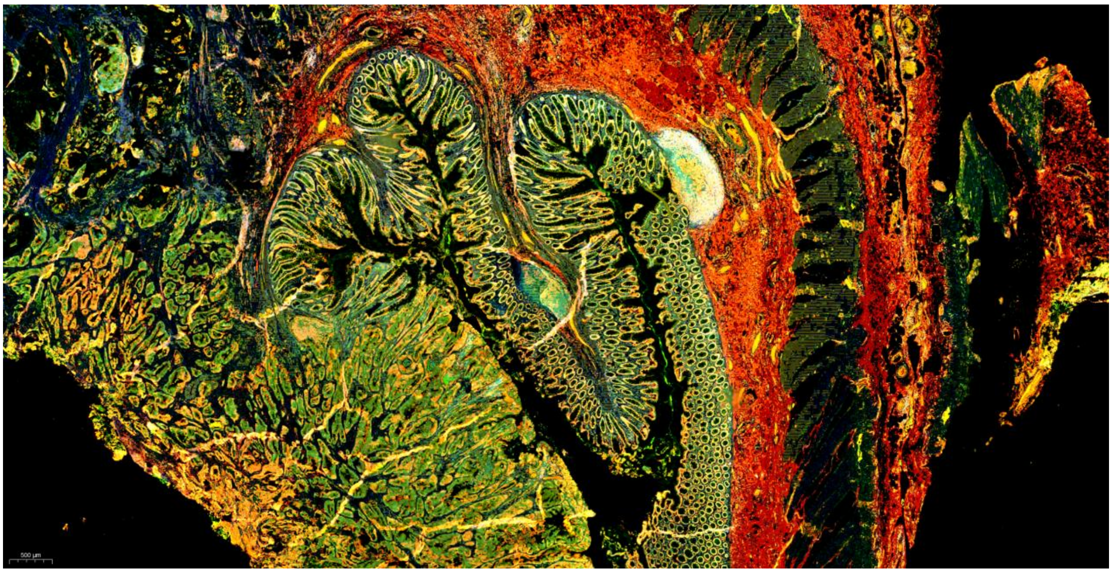
500 µm

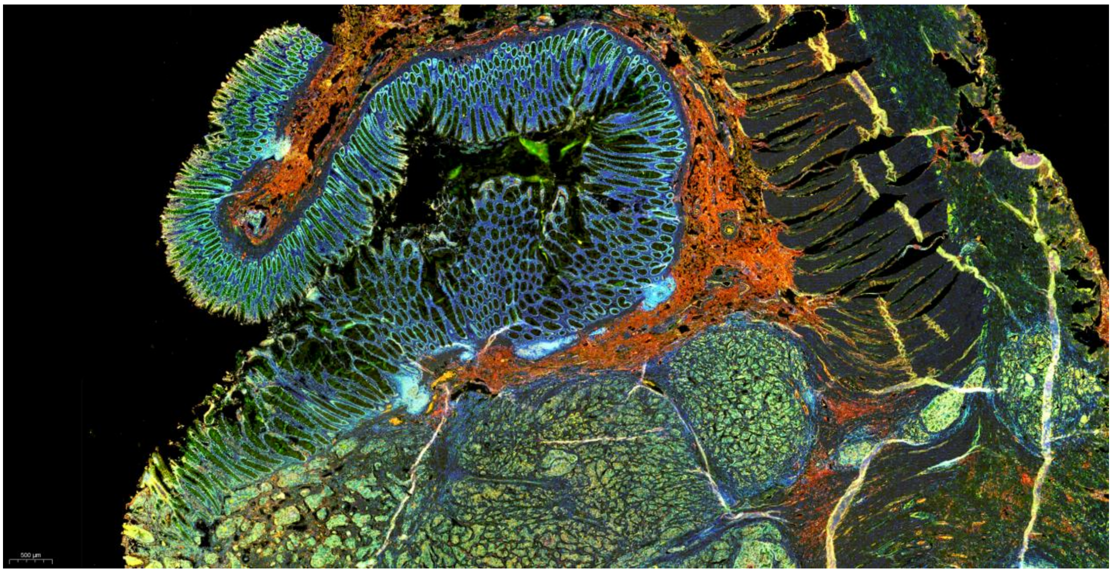
500 µm

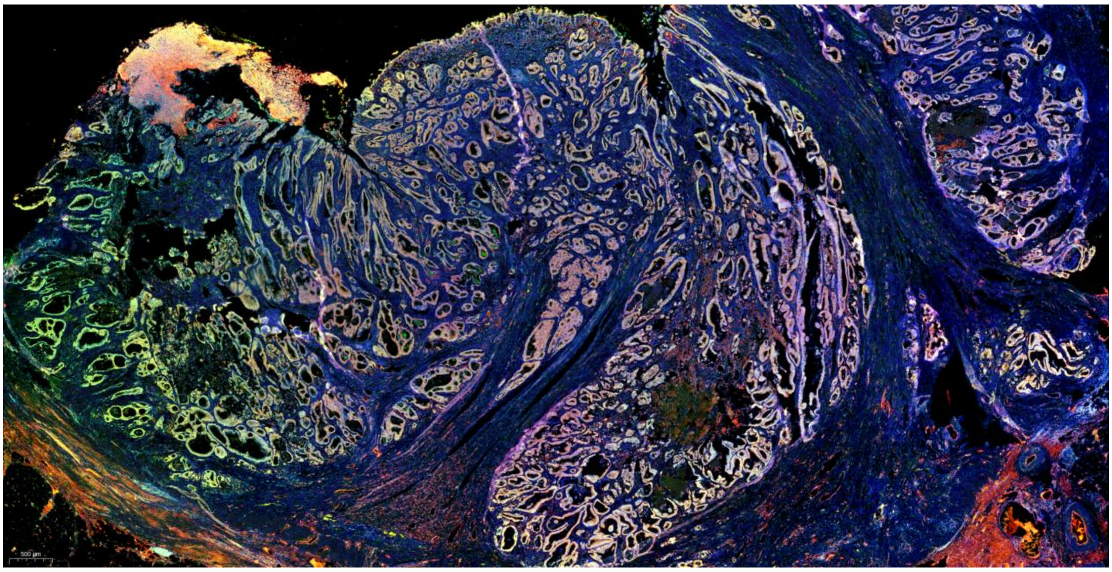
500 µm

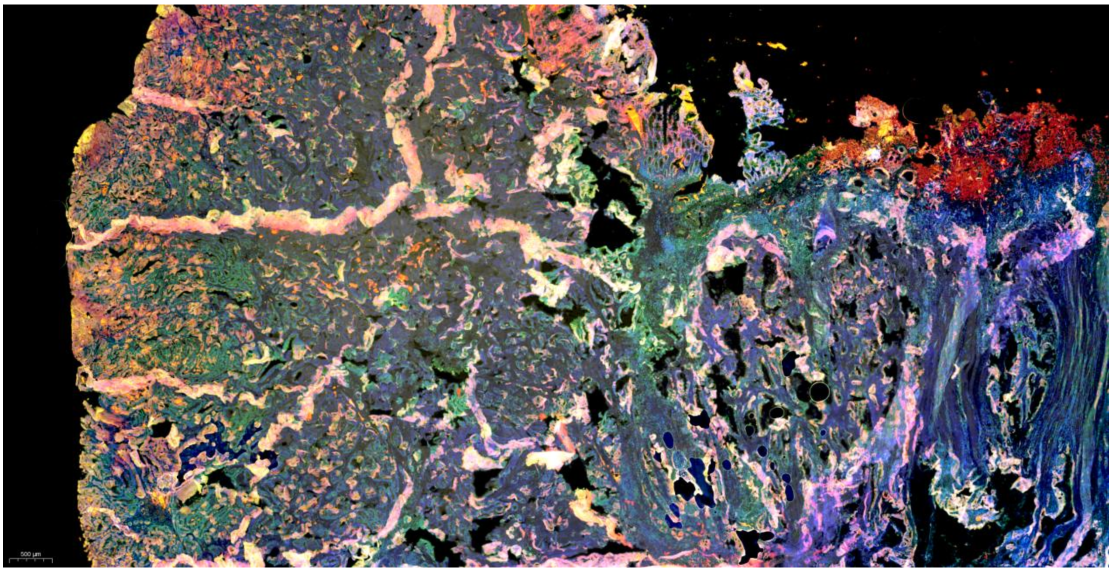
500 µm

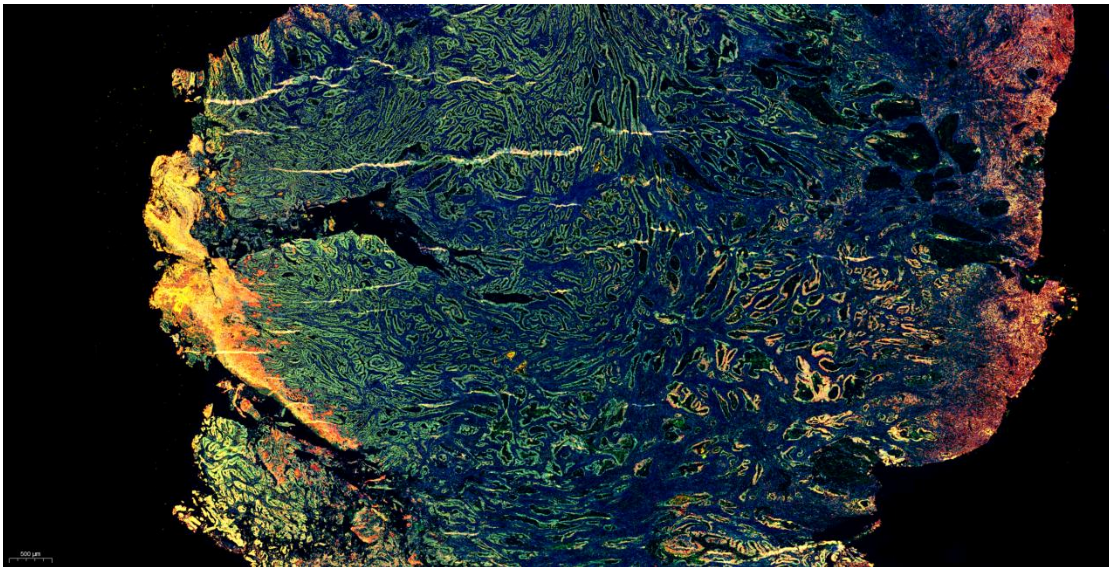
500 µm

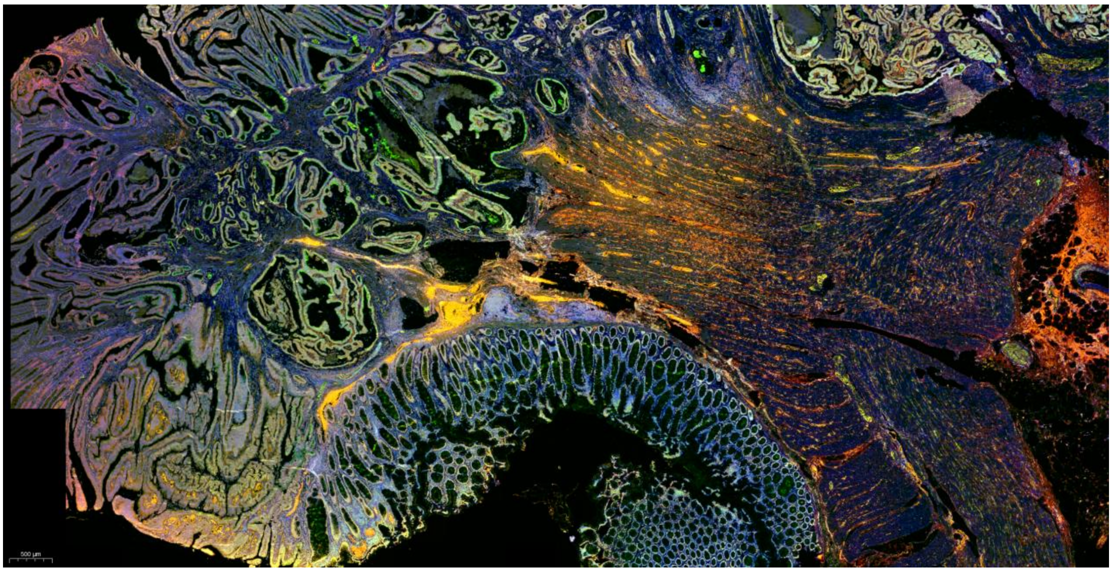
500 µm

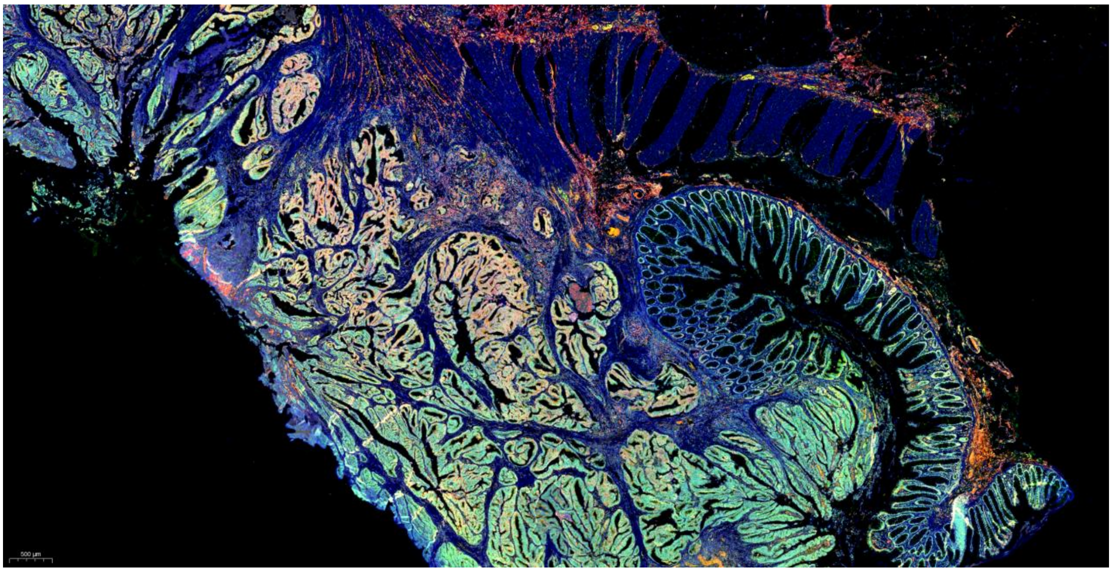
500 µm

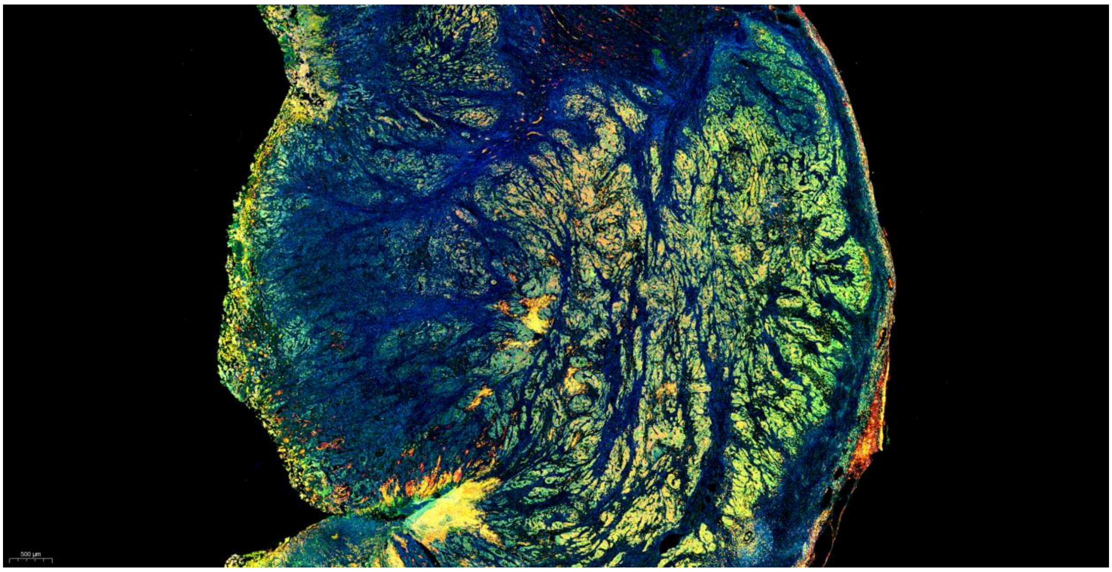
500 µm

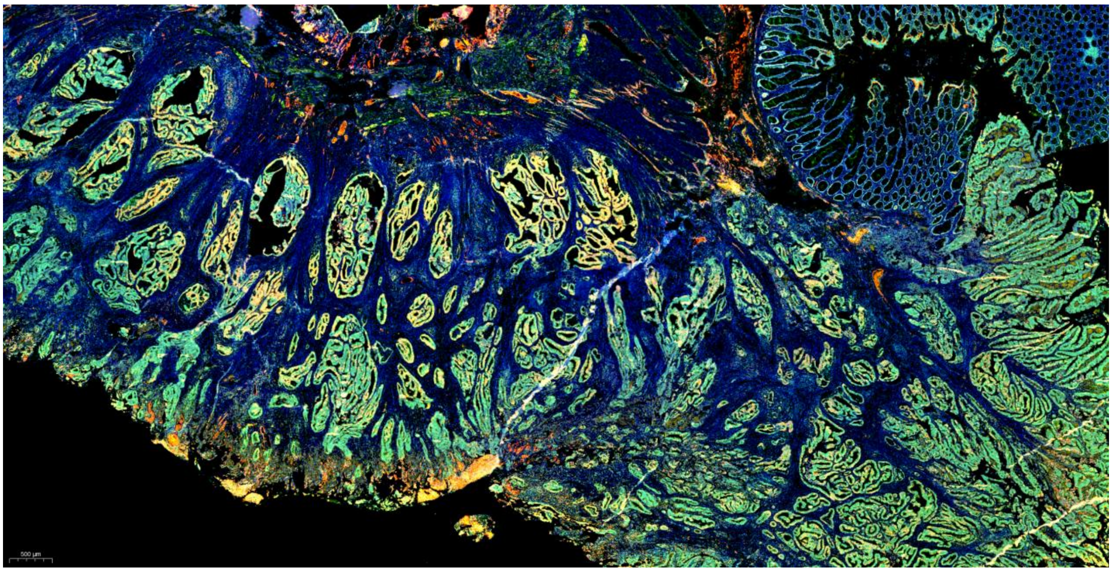
500 µm

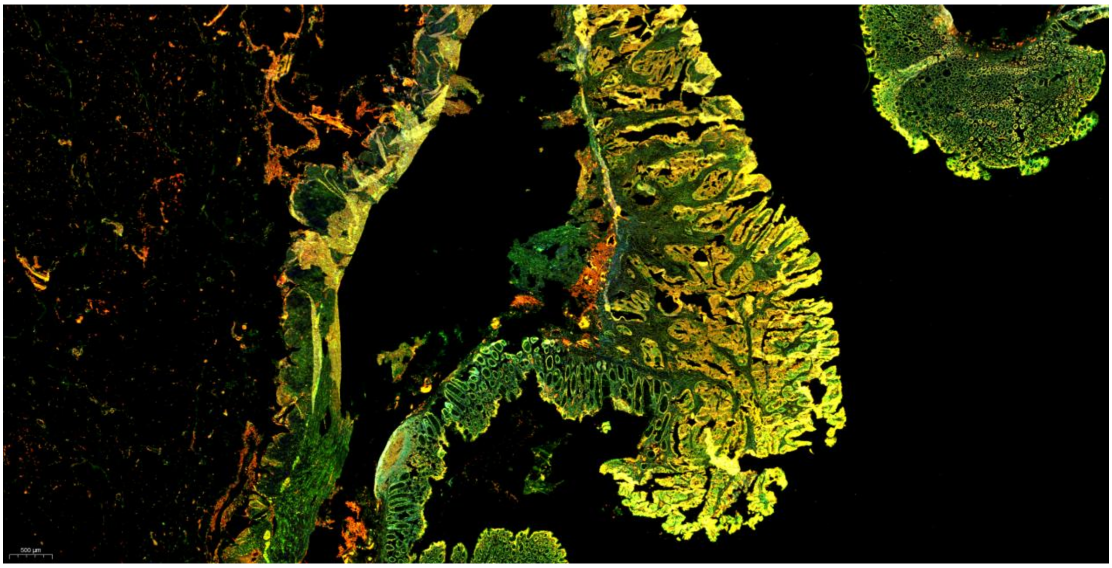
500 µm

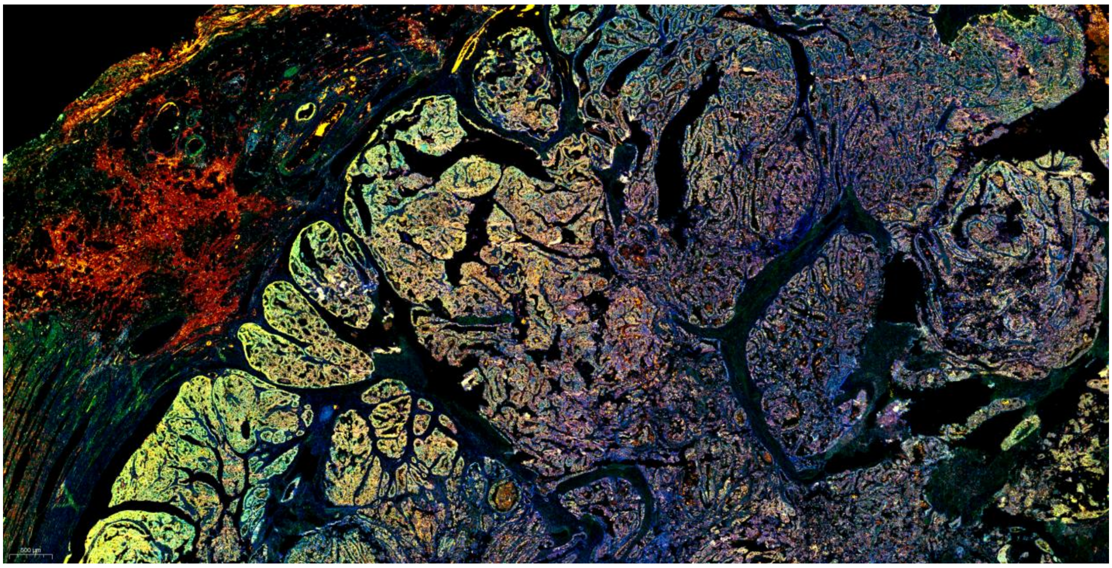
500 µm

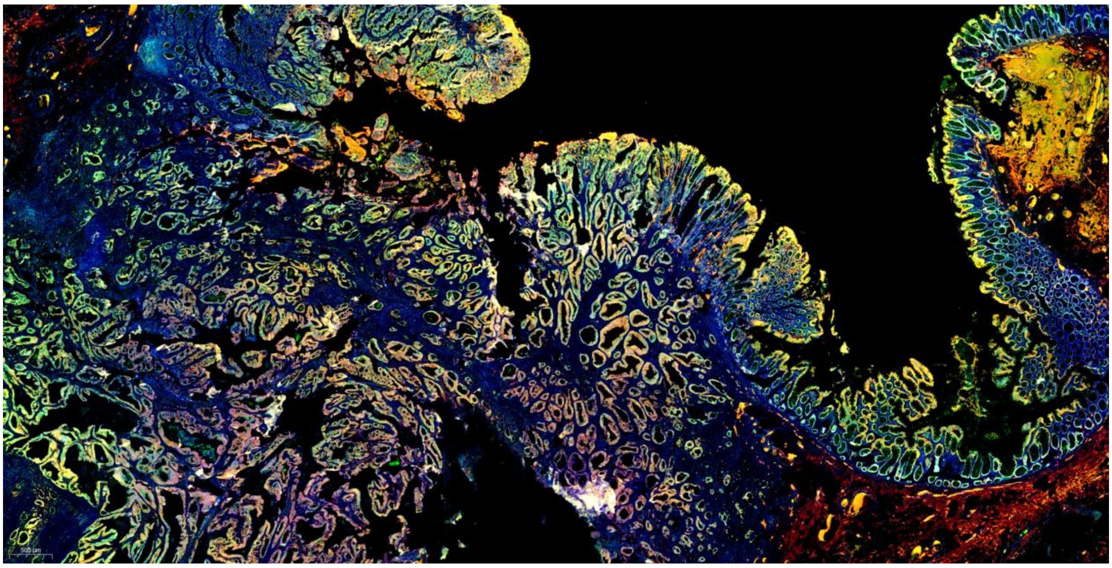
500 µm

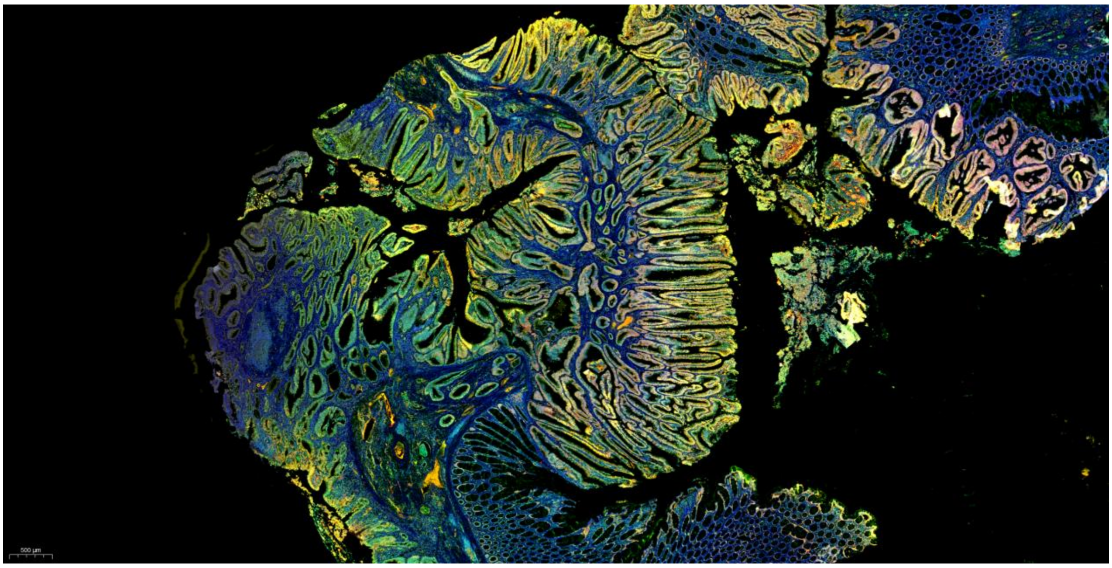
500 µm

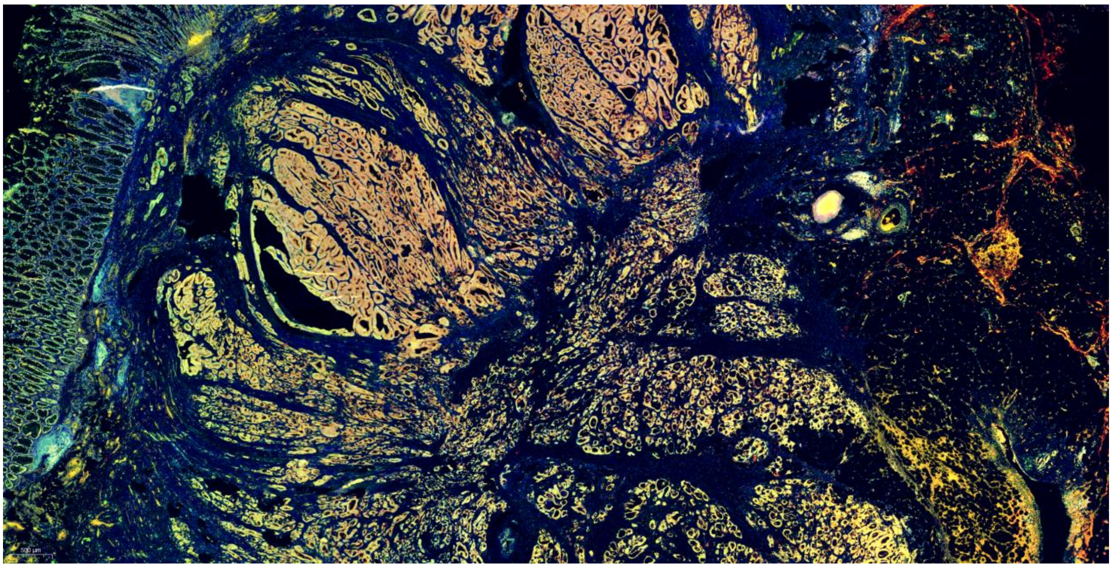
500 µm

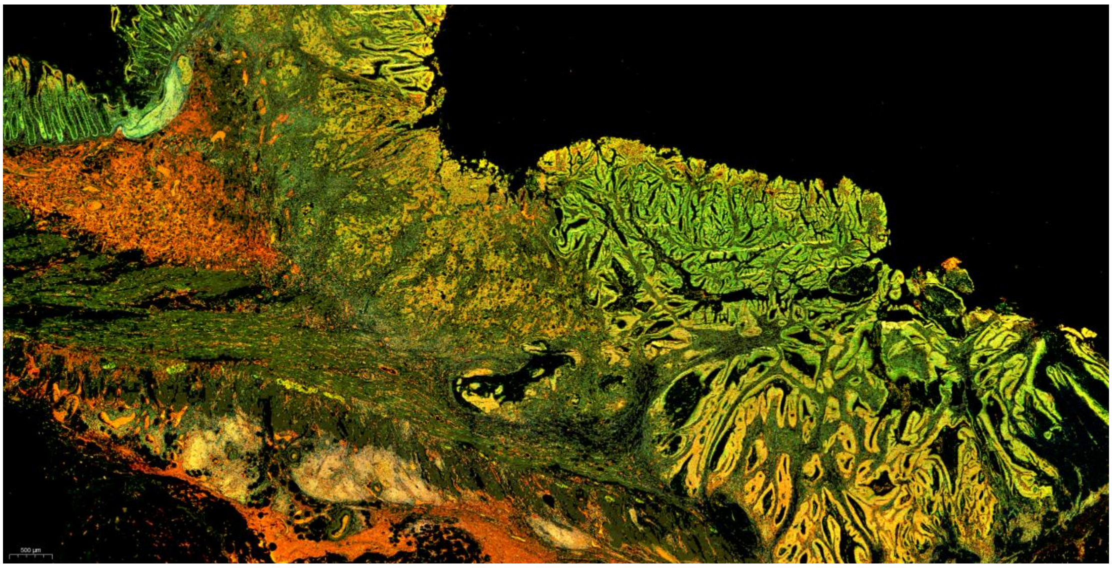
500 µm

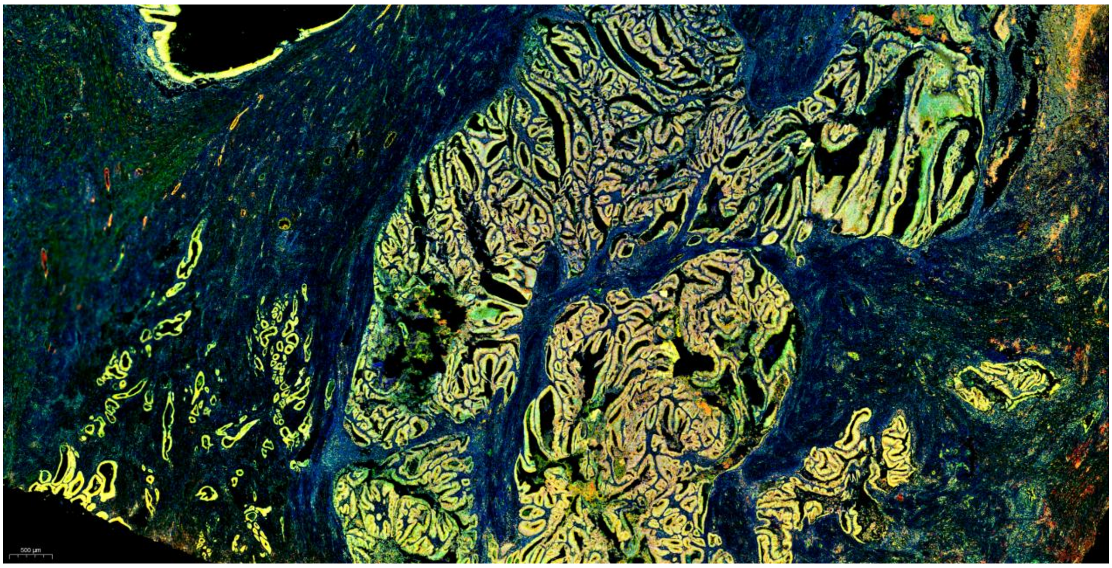
500 µm

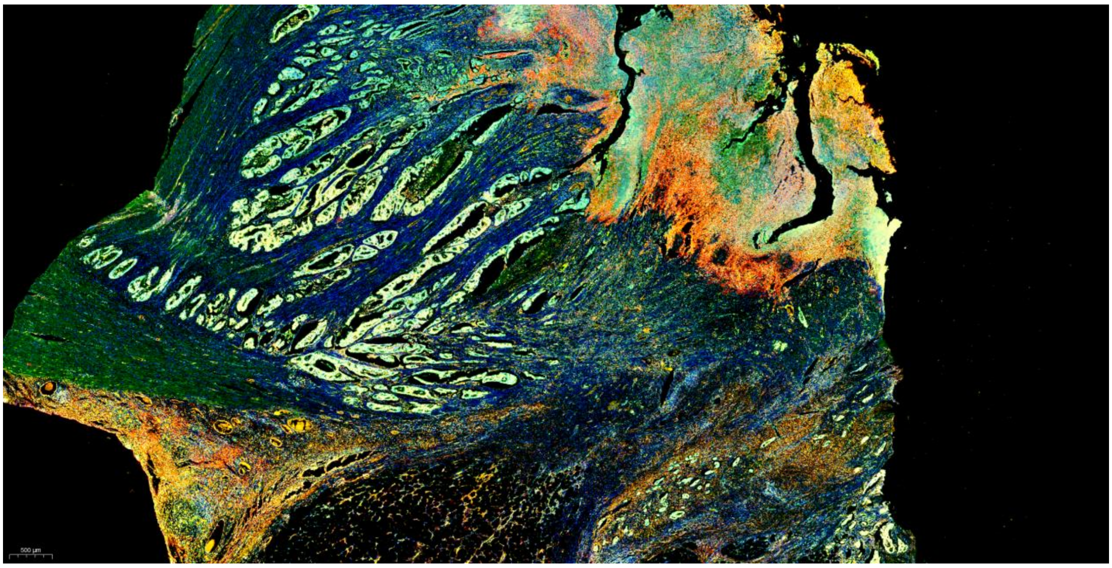
500 µm

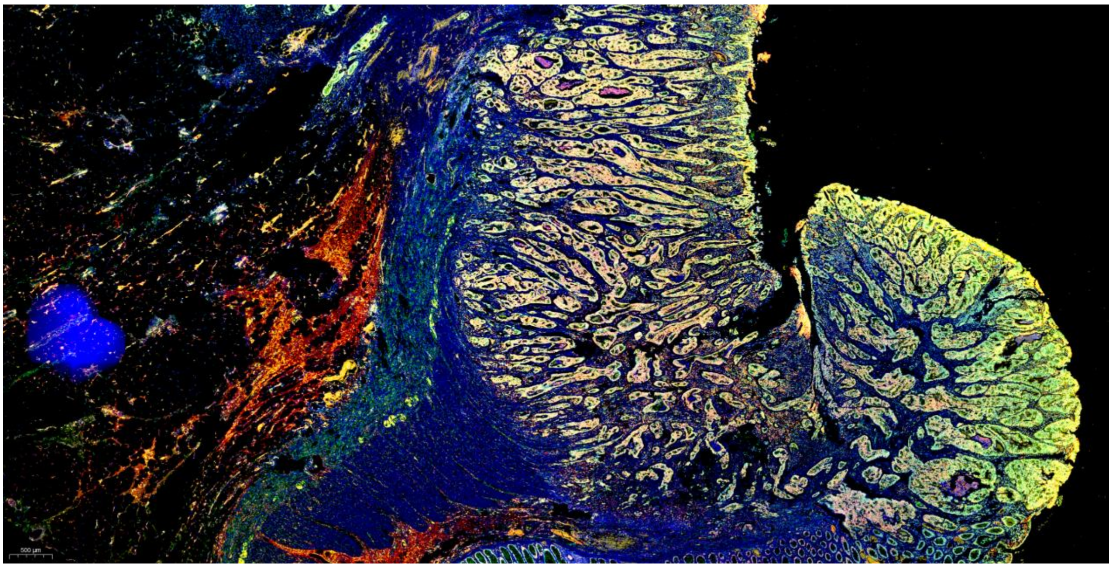

Red: WNT4; Green: IGF2; Yellow: STAT3
